# Diagnostic Value of Psychomotor Vigilance Task for Severe Sleep Inertia in Idiopathic Hypersomnia Versus Other Sleep Disorders Without Sleep Inertia

**DOI:** 10.1111/jsr.70377

**Published:** 2026-05-31

**Authors:** Elisa Evangelista, Alexandre Derré, Lucie Barateau, Anna Laura Rassu, Sofiene Chenini, Isabelle Jaussent, Yves Dauvilliers

**Affiliations:** ^1^ Sleep Unit, CHU Nîmes Nîmes France; ^2^ National Reference Centre for Orphan Diseases Narcolepsy Rare Hypersomnias, Sleep Unit, Department of Neurology, CHU Montpellier Montpellier France; ^3^ Institute for Neurosciences of Montpellier INM, Univ Montpellier, INSERM Montpellier France

**Keywords:** idiopathic hypersomnia, lapses, psychomotor vigilance task, sleep inertia

## Abstract

Sleep inertia impairs performance upon awakening and may be assessed using the psychomotor vigilance task (PVT). This study aimed to determine optimal PVT lapse cut‐offs at awakening to objectively measure sleep inertia by comparing patients with idiopathic hypersomnia (IH) and severe sleep inertia to patients with other sleep disorders (OSD) without sleep inertia, to assess the discriminative performance of these thresholds in OSD patients with sleep inertia; and to explore factors associated with PVT lapses. 350 drug‐free consecutive patients (227 females, 30.7 ± 11.0 y.o.) were included: 131 IH and 219 OSD (75 non‐specified hypersomnolence, 54 narcolepsy, 50 obstructive sleep apnea, 40 others). All patients performed two 5‐min PVTs at 07:00 pm and 07:00 am upon awakening. Sleep inertia was self‐reported with the Idiopathic Hypersomnia Severity Scale. Receiver operating characteristic analyses were used to determine optimal cut‐offs for 7:00 am PVT lapses and the change between 07:00 am and 07:00 pm in IH patients with severe sleep inertia (*n* = 73) and OSD patients without sleep inertia (*n* = 84). Optimal PVT cut‐offs were ≥ 4 for 7 am‐Lapses (sensitivity 72.6%, specificity 69.1%) and ≥ 4 for Delta 7 am‐7 pm‐Lapses (sensitivity 50.9%, specificity 83.3%). Across all 350 participants, 56.9% exhibited 7 am‐Lapses ≥ 4, while 39.7% had Delta 7 am‐7 pm Lapses ≥ 4. In conclusion, a threshold of ≥ 4 PVT lapses at 07:00 am, along with an increase of ≥ 4 lapses from 07:00 pm to 07:00 am best capture sleep inertia–related performance impairment in IH, supporting their use as objective markers in clinical practice and research settings.

## Graphical abstract

Diagnostic Value of PVT for Severe Sleep Inertia in Idiopathic Hypersomnia.

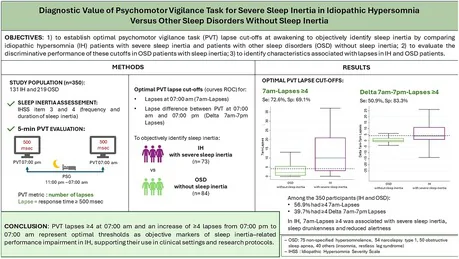

## 1 Introduction

Sleep inertia is a transient state characterized by impaired performance and reduced alertness during the transition from sleep to wakefulness (Trotti 2017; Ruby et al. 2024). It reflects a delay in the functional reorganization of brain networks upon awakening, leading to slow reactivation of networks related to alertness and higher‐order cognitive processes (Tsai et al. 2014; Balkin et al. 2002; Vallat et al. 2019). The extent of the performance impairment upon awakening is highly variable and may cause serious alterations in tasks that require attention and decision‐making (Hilditch and McHill 2019; Horne and Moseley 2011).

Sleep inertia is a common phenomenon observed in the general population (Ohayon et al. 2000; Ohayon et al. 2014). However, a severe form of sleep inertia may be found in sleep disorders (Trotti 2017; Ruby et al. 2024; Ohayon et al. 2000) and is one of the key features of idiopathic hypersomnia (IH), together with excessive daytime sleepiness (EDS) and excessive quantity of sleep (EQS) (American Academy of Sleep Medicine 2023; Lammers et al. 2020; Billiard and Dauvilliers 2001; Arnulf et al. 2019). Up to 57% of patients with IH demonstrated a severe and disabling sleep inertia (Anderson et al. 2007; Vernet and Arnulf 2009; Bassetti and Aldrich 1997; Evangelista et al. 2018; Evangelista et al. 2022). Recently, we reported the high frequency and intensity of severe sleep inertia after nighttime sleep in IH, using three items from the validated Idiopathic Hypersomnia Severity Scale (IHSS) (Evangelista et al. 2022; Dauvilliers et al. 2019). Using the psychomotor vigilance task (PVT), one of the most widely used tools for assessing behavioural alertness and sustained attention, we also found that patients with IH who reported severe sleep inertia showed a marked increase in the number of lapses (i.e., response time, RT, ≥ 500 msec) upon awakening (Tkachenko and Dinges 2018). Importantly, PVT results did not differ between patients with severe sleep inertia in IH and those with severe sleep inertia in other sleep disorders. However, the presence and duration of sleep inertia, and the number of PVT lapses varied considerably among patients (Evangelista et al. 2022). IH is a disabling neurological disorder of hypersomnolence with heterogeneous presentation. It has been described as comprising two clinical phenotypes: one with long sleep time (≥ 10 h/night), and one without long sleep time (< 10 h/night) in the ICSD, 2nd Edition (ICSD‐2) (American Academy of Sleep Medicine 2005). However, more recent editions, including the current one (ICSD3‐TR) (American Academy of Sleep Medicine 2023), have grouped IH under a single definition based on a mean sleep latency ≤ 8 min on multiple sleep latency test (MSLT), and/or total sleep time (TST) ≥ 11 h per 24 h, assessed by 24‐h polysomnography monitoring or estimated by wrist actigraphy combined with a sleep log (averaged over at least seven days with unrestricted sleep). Sleep inertia is a supportive clinical feature of IH, being not mandatory for its diagnosis (American Academy of Sleep Medicine 2023).

To the best of our knowledge, no study has yet defined a threshold for the number of PVT lapses that can be used to correlate the performance impairment observed in patients with IH and severe self‐reported sleep inertia. Furthermore, the underlying mechanisms and the characteristics associated with severe sleep inertia in IH and more generally in sleep disorders remain poorly understood (Trotti 2017; Ruby et al. 2024).

The aims of this study were to determine the optimal cut‐off for PVT lapses upon awakening at 07:00 am and for the difference in lapses between 07:00 am and 07:00 pm, to (1) objectively measure sleep inertia by comparing idiopathic hypersomnia (IH) patients with severe sleep inertia to those with other sleep disorders (OSD) without sleep inertia; (2) the discriminative performance of these thresholds in OSD patients with sleep inertia; and (3) explore the clinical and polysomnographic characteristics associated with these measures in the overall population, and then separately in patients with IH and OSD.

## 2 Methods

### 2.1 Patients

Between 2016 and 2023, 350 consecutive drug‐free participants aged over 16 underwent a standardized evaluation including polysomnography (PSG), self‐reported sleep inertia assessment using the IHSS, and two 5‐min PVT sessions (one at 07:00 pm before PSG and one at 07:00 am the following morning), at the Sleep Unit and National Reference Center for Rare Hypersomnia Disorders in Montpellier, France. Participants had no significant comorbid conditions other than sleep disorders and were either drug‐naïve (*n* = 259) or had stopped taking medications that could affect sleep–wake patterns for at least two weeks (*n* = 91).

Patients were diagnosed with IH based on ICSD‐3 TR criteria (American Academy of Sleep Medicine 2023) and alternative validated criteria (Evangelista et al. 2018). Specifically, patients had to report EQS and/or EDS, and fulfil either the ICSD‐3 TR MSLT criterion, namely mean sleep latency ≤ 8 min with < 2 sleep‐onset rapid eye movement (REM) periods (SOREMPs) and/or the alternative total sleep time criterion, defined as ≥ 11 h over 24 h or a TST ≥ 19 h on the 32‐h bed‐rest PSG recording (Evangelista et al. 2018). The 32‐h bed‐rest recording (Night 1 [11:00 pm–7:00 am] + Daytime [07:00 am–11:00 pm] + Night 2 [11:00 pm–07:00 am]) is designed to obtain the maximal spontaneous amount of sleep under standardized conditions, avoiding external influences, and is preceded by a PSG—modified MSLT (i.e., forced awakening after 1 min of sleep) to minimize interference with the sleep homeostatic process. Most of patients with a highly probable diagnosis of IH underwent this protocol and were then classified as having or not a long sleep time (i.e., TST ≥ 19 h/32). By definition, patients with IH did not have an apnea‐hypopnea index (AHI) ≥ 15/h, a nighttime TST on PSG < 6 h, sleep deprivation (i.e., < 7 h of sleep per night based on sleep diary), night or shift work, abnormal advanced or delayed sleep–wake phase disorders (i.e., ≥ 2 h delay relative to the required or desired sleep period), and cataplexy (American Academy of Sleep Medicine 2023).

Patients with other sleep disorders (OSD) were diagnosed with narcolepsy type 1 (NT1; all with EDS, typical cataplexy, and cerebrospinal fluid hypocretin‐1 < 110 pg/mL), obstructive sleep apnea syndrome (OSAS: AHI ≥ 5/h), non‐specified hypersomnolence (NSH; complaint of EDS and/or EQS, AHI < 5/h and TST ≥ 6 h on the PSG, but without meeting the diagnostic criteria for a central disorder of hypersomnolence). Also, patients with chronic insomnia, restless legs syndrome and/or short TST (i.e., < 6 h) on the PSG were categorized as “others” sleep disorders.

This study was approved by the local ethics committees (Comité de Protection des Personnes, France: “Constitution of a cohort and of a clinical, neurophysiological and biological bank of hypersomnolence disorders”—SOMNOBANK). Consent to participate was provided by all patients and by both parents for < 18‐year‐old adolescents.

### 2.2 Clinical Evaluation

All participants underwent a comprehensive clinical assessment including body mass index (BMI) (<25 kg/m2: thin/normal, ≥25 and < 30 kg/m2: overweight, ≥30 kg/m2: obesity), detailed medical history, and sleep disorder screening by sleep experts. They completed the Epworth Sleepiness Scale (ESS; score > 10: EDS) (Johns 1991), IHSS (score ≥ 26: severe symptoms) (Dauvilliers et al. 2019; Rassu et al. 2022) and Beck Depression Inventory II (BDI‐II; score ≤ 13: minimal, 14–19: mild, 20–63: moderate–severe depressive symptoms) (Beck et al. 1996). Participants' habitual bedtime and wake‐up time were also assessed.

Self‐reported sleep inertia during the month preceding PSG was assessed using items 3 and 4 of the IHSS: “Is it extremely difficult for you, or even impossible, to wake up in the morning without several alarm calls or the help of someone?” (score 3: always, 2: often, 1: sometimes, 0: never), and “After a night sleep, how long does it take to feel like you are functioning properly (i.e., fully functional, both physically and intellectually) after getting out of bed?” (score 4: ≥ 2 h; 3: > 1 h but < 2 h; 2: between 30 min and 1 h; 1: < 30 min; 0: I feel I am functioning properly as soon as I wake up) (Evangelista et al. 2022; Dauvilliers et al. 2019). Sleep inertia was defined as severe when scores were ≥ 2 on both items, and mild otherwise. Sleep drunkenness was defined by a score ≥ 2 on IHSS item 5: “In the minutes after waking up, do you ever do irrational things and/or say irrational things, and/or are you very clumsy, for example, tripping, breaking or dropping things?” (score 3: always, 2: often, 1: sometimes, 0: never).

Two additional IHSS items were used to assess night‐time sleep need (item 1: “What for you is the ideal duration of night‐time sleep at the weekend or on holiday”; score 4: ≥ 11 h, 3: > 9 h and < 11 h, 2: between 7 h and 9 h, 1: < 7 h), and hypersomnolence‐related driving impairment (item 14: “Do you consider that your hypersomnolence is a problem in terms of your driving a car?”; score 4: very significant, 3: significant, 2: moderate, 1: minor, 0: no problem, I do not drive).

### 2.3 Neurophysiological Evaluation

The standard PSG recording (11:00 pm–07:00 am) included electroencephalogram leads, electrooculogram, chin electromyogram, cannula/pressure transducer system, mouth thermistor, chest and abdominal bands, pulse oximeter, electromyogram electrodes on bilateral anterior tibialis muscles, and electrocardiogram. Sleep variables included sleep duration, sleep efficiency, percentage of N1, N2, N3 and REM sleep, sleep latency, REM sleep latency, wake after sleep onset (WASO), periodic leg movements during sleep (PLMS), micro‐arousal indices and AHI (Iber et al. 2007; Ferri et al. 2016). Sleep apnea was defined as a drop in peak flow signal excursion by ≥ 90% for ≥ 10 s, and hypopnea as a drop in the peak flow signal excursion by ≥ 30% for ≥ 10 s associated with ≥ 3% desaturation and/or a micro‐arousal (Berry et al. 2012). Sleep‐related respiratory events were classified as obstructive, central or mixed and stratified into mild (AHI = 5–14.9/h), moderate (AHI = 15–29.9/h), and severe (AHI ≥ 30/h). A MSLT following the PSG was conducted in 307 (88%) patients complaining of EDS.

The percentages of wake, N1, N2, N3 and REM sleep during the last hour of PSG (i.e., 06:00‐:00 am, just before the morning PVT session) were also calculated.

### 2.4 Psychomotor Vigilance Task

Two PVT sessions were performed in the laboratory, one at 07:00 p.m. before PSG (on the first night for patients undergoing multiple nights of recording), and the other at 07:00 a.m. immediately upon awakening, using the short 5‐min PVT version (Evangelista et al. 2022; Basner et al. 2011; Basner and Dinges 2011; Loh et al. 2004). All patients were awakened at 07:00 a.m. by an investigator to perform the PVT in a standardized manner, with the lights on and the patient seated on the bed. During the PVT, subjects were instructed to respond to randomly presented visual stimuli (red millisecond‐counter on a computer screen) by pressing a button as quickly as possible. Primary PVT outcomes were the number of lapses at 07:00 a.m. (7 a.m.‐Lapses) and the difference in the number of lapses between 07:00 a.m. and 07:00 p.m. (Delta 7 a.m.‐7 p.m.‐Lapses). Secondary outcomes included the mean RT, median RT, mean 1/RT, slowest 10% 1/RT, fastest 10% RT, and number of lapses + false starts (false start = RT < 100 msec), both at 07:00 a.m. and as differences between 07:00 a.m. and 07:00 p.m. Subjective alertness was assessed just before the 07:00 a.m. PVT session using the Karolinska Severity Scale (KSS) (from 1 = very alert, to 9 = very sleepy), French version (Akerstedt and Gillberg 1990).

### 2.5 Statistical Analysis

The sample was described using numbers and percentages for categorical variables, and means with standard deviations (SD) or medians with ranges for continuous variables, as appropriate. Differences in 7 am‐Lapses between IH patients with severe sleep inertia and OSD patients without sleep inertia were assessed using logistic regression models. Receiver Operating Characteristic (ROC) curves were used to identify optimal PVT cut‐off values for 7 am‐Lapses and Delta 7 am‐7 pm‐Lapses to best discriminate the presence of sleep inertia, using IH patients with severe sleep inertia and OSD patients without sleep inertia as reference groups. Optimal cut‐offs were defined by the highest Youden index, calculated as [specificity + sensitivity]−1. Internal validation of the cut‐offs was performed using a bootstrap procedure with 200 replications to ensure robustness.

To examine whether additional PVT metrics improved discrimination beyond lapses, AUCs for lapses were compared with those of other PVT metrics (mean RT, median RT, mean 1/RT, slowest 10% 1/RT, fastest 10% RT, and number of lapses + false starts) using DeLong's test for correlated ROC curves.

Associations between demographic, clinical, and PSG characteristics and PVT outcomes were analysed using logistic regression models (1) in the whole sample, (2) in IH patients, and (3) in OSD patients, based on the optimal cutoffs for the following PVT metrics: 7 am‐Lapses, Delta 7 am‐7 pm‐Lapses, 7 am‐mean 1/RT, Delta 7 am‐7 pm‐mean 1/RT. As this was an exploratory study, no adjustments for multiple comparisons were made to avoid a conservative approach that could mask potentially important associations. The significance level was set at *p* < 0.05. Analyses were performed using SAS (v9.4; SAS, Cary, NC).

## 3 Results

A total of 350 drug‐free participants were included (mean age: 30.75 ± 10.97 years; 227 females). Among them, 131 had IH (mean age: 27.75 ± 9.00 years; 108 females), of whom 67 (51.1%) had a mean sleep latency ≤ 8 min on the MSLT. Most IH patients (121, 92.4%) underwent a 32‐h bed‐rest recording, and 106 (87.6%) met criteria for long sleep time (total sleep time ≥ 19 h/32; all ≥ 11 h/24). Within the IH group, 73 patients (55.7%; mean age: 27.22 ± 7.97 years), including 62 with the long sleep time phenotype, reported severe sleep inertia based on IHSS items 3–4. This subgroup comprised 63 women (58.3%) and 10 men (43.5%); 20 reported no sleep inertia and 34 mild sleep inertia (Table 1). The remaining 219 participants (mean age: 32.55 ± 11.65 years; 119 females) had other sleep disorders (OSD), including 54 with narcolepsy type 1 (NT1), 75 with NSH, 50 with obstructive sleep apnea syndrome (OSAS; 31 mild, 13 moderate, 6 severe), and 40 with other conditions. Among these OSD patients, 84 (38.4%; 37 females; mean age: 35.16 ± 12.03 years) reported no sleep inertia, 90 (41%) reported severe sleep inertia, and the remainder reported mild sleep inertia (Table 1). Overall, 202 of the 350 participants had been included in a previous study (Evangelista et al. 2022).

**TABLE 1:** Demographic and clinical characteristics of all study patients and by diagnostic group (IH vs. OSD).

	All patients *N* = 350	OSD *N* = 219	IH *N* = 131	*p*
Age (years)^a^	30.75 (±10.97)	32.55 (±11.65)	27.75 (±9.00)	0.0001
Sex, female^b^	227 (64.86)	119 (54.34)	108 (82.44)	< 0.0001
BMI (kg/m^2^)^a^	23.96 (±4.79)	24.69 (±4.97)	22.75 (±4.22)	0.0003
Treatment status, Naïve^b^	259 (74.00)	169 (77.17)	90 (68.70)	0.08
ESS score^a^	14.47 (±5.09)	13.91 (±5.50)	15.41 (±4.16)	0.009
ESS score, > 10^b^	272 (78.61)	157 (72.69)	115 (88.46)	0.0008
Total IHSS score^a^	30.13 (±8.65)	28.27 (±8.87)	33.13 (±7.37)	< 0.0001
Total IHSS score, ≥ 26^b^	240 (74.53)	134 (67.34)	106 (86.18)	0.0002
Partial IHSS score^a^	25.65 (±7.07)	24.22 (±7.45)	27.96 (±5.74)	< 0.0001
Self‐reported sleep inertia^b^				< 0.0001
Absent	104 (29.71)	84 (38.36)	20 (15.27)	
Mild	83 (23.71)	45 (20.55)	38 (29.01)	
Severe without drunkenness	99 (28.29)	62 (28.31)	37 (28.24)	
Severe with drunkenness	64 (18.29)	28 (12.79)	36 (27.48)	
Driving impairment, Moderate to very significant^b^	147 (42.00)	78 (35.62)	69 (52.67)	0.002
Night‐time sleep needs, ≥ 9 h^b^	209 (65.93)	109 (55.90)	100 (81.97)	< 0.0001
Habitual bedtime (hh:mm)^a^	22:59 (±1:04)	22:56 (±1:06)	23.01 (±1:01)	0.52
Habitual wake up time (hh:mm)^a^	08:03 (±1:51)	07:55 (±1:47)	08:13 (±1:55)	0.17
BDI^a^	14.00 (±9.60)	13.99 (±9.14)	14.00 (±10.21)	0.99
BDI‐II score^b^				0.86
< 14	157 (53.22)	87 (52.10)	70 (54.69)	
14–20	57 (19.32)	34 (20.36)	23 (17.97)	
≥ 20	81 (27.46)	46 (27.54)	35 (27.34)	
7:00‐KSS score^a^	6.31 (±2.05)	5.95 (±2.02)	6.91 (±1.97)	0.009

Abbreviations: BDI‐II = Beck Depression Inventory II, BMI = body mass index, ESS = Epworth Sleepiness Scale, IHSS = Idiopathic Hypersomnia Severity Scale, KSS = Karolinska Sleepiness Scale.

^a^ Quantitative variables are expressed as means (±standard deviation).

^b^ Categorical variables are shown as *n* (%).

Compared to OSD patients without sleep inertia (*n* = 84), IH patients with severe sleep inertia (*n* = 73) were younger and more frequently female, had lower BMI, and higher scores on ESS, total IHSS and partial IHSS (i.e., after exclusion of the three items related to sleep inertia and sleep drunkenness), BDI‐II and 7:00‐KSS. They also reported higher driving impairment and night‐time sleep needs (ideal sleep duration ≥ 9 h/night), and later habitual wake‐up time. Moreover, they had higher sleep duration and sleep efficiency, along with lower N1, WASO, micro‐arousal, PLMS and AHI indices on the PSG, and higher N2 and REM with reduced wakefulness and N1 during the last hour of the PSG recording.

### 3.1 Optimal PVT Lapse Threshold to Objectively Measure Severe Sleep Inertia in IH Patients Compared With Those With Other Sleep Disorders Without Sleep Inertia

The number of 7 am‐Lapses was higher in IH patients with severe sleep inertia (*n* = 73) compared to OSD patients without sleep inertia (*n* = 84) (mean ± SD: 12.73 ± 11.29 vs. 3.93 ± 6.07; median [range]: 10.00 [0.00;37.00] vs. 2.00 [0.00;35.00], *p* < 0.0001). A cut‐off of ≥ 4 for 7 am‐lapses was the most effective for identifying severe sleep inertia in IH compared with OSD patients without sleep inertia, with a sensitivity of 72.6%, and a specificity of = 69.1% (AUC = 78%, 95% CI = [70–85]) (Figure 1, Figure S1, Table S1). Similarly, Delta 7 am‐7 pm‐Lapses was higher in IH patients with severe sleep inertia compared to OSD patients without sleep inertia (6.64 ± 9.82 vs. 1.38 ± 4.44; 4.00 [−31.00;28.00] vs. 0.00 [−7.00;26.00], *p* = 0.0002), with an optimal discriminative threshold of ≥ 4 (Delta 7 am‐7 pm‐Lapses: AUC = 74%, 95% CI = [66–82]; sensitivity = 56.2%, specificity = 83.3%). Both AUCs for mean 7 am‐Lapses and Delta 7 am‐7 pm remained consistent after internal validation using bootstrap sampling with 200 replications.

**FIGURE 1:**
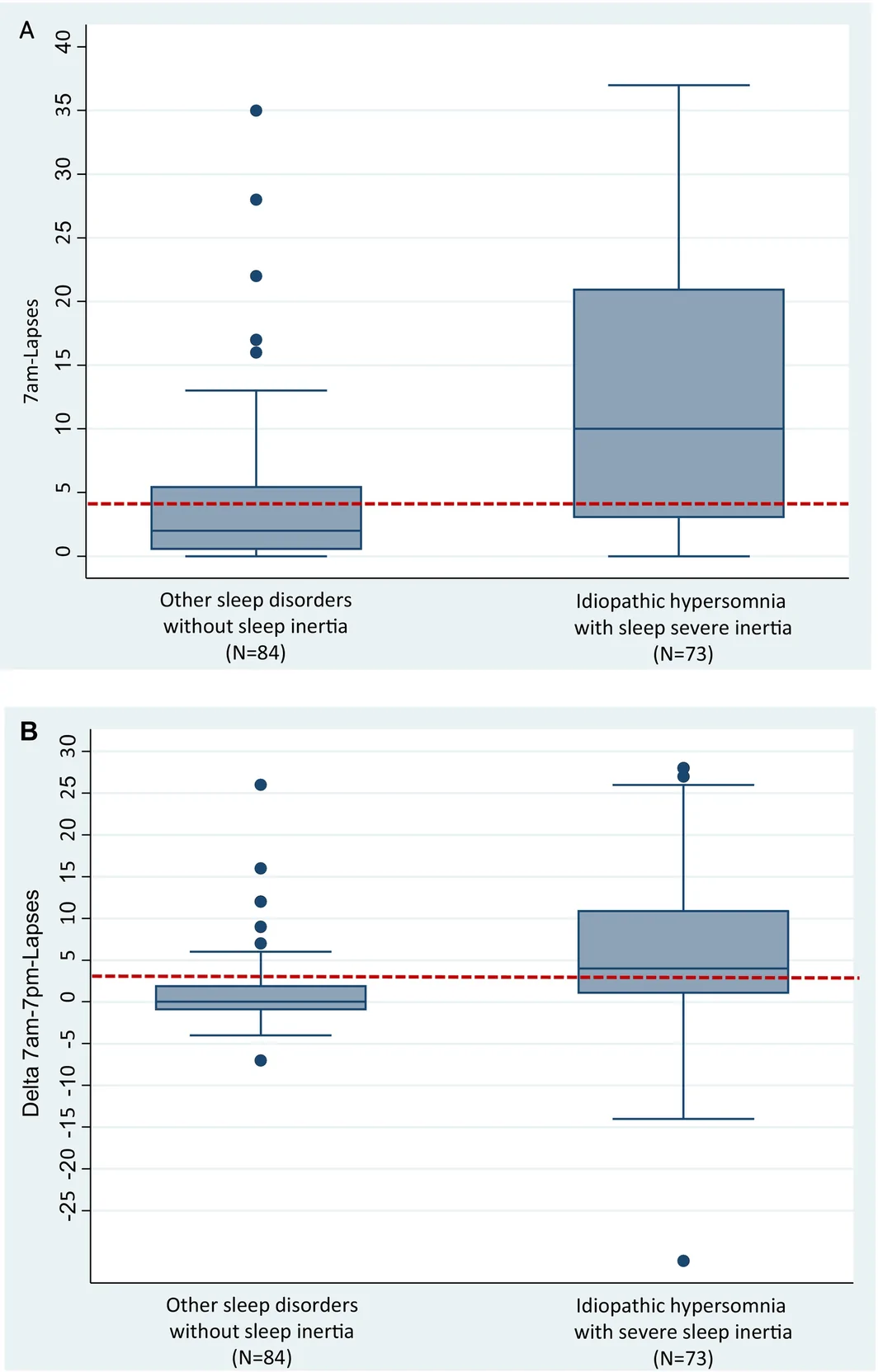
Distribution of the number of PVT lapses at 07:00 am (7 am‐Lapses) (A), and the difference in PVT lapse number between 07:00 am and 07:00 pm the day before (Delta 7 am‐7 pm‐Lapses) (B), in patients with IH and severe sleep inertia (*n* = 73) and in patients with other sleep disorders without sleep inertia (*n* = 84). Box plots show the median value, 25th‐75^th^ percentiles and range. Dots indicate out‐of‐range values. The dashed lines represent the cut‐off values: The cut‐off of 4 for the 7 am‐Lapses displayed a sensitivity of 72.6% and a specificity of 69.1%, the cut‐off of 4 for the Delta 7 am‐7 pm‐Lapses displayed sensitivity of 56.2% and a specificity of 83.3%.

Applying the thresholds of ≥ 4 for both 7 am‐Lapses and Delta 7 am‐7 pm‐Lapses across all patients with severe sleep inertia (*n* = 163: *n* = 73 IH, *n* = 11 NT1, *n* = 45 NSH, *n* = 20 OSAS, *n* = 14 others) versus OSD patients without sleep inertia (*n* = 84), produced similar results (7 am‐Lapses: AUC = 75%, 95% CI = [69–81], sensitivity = 68.1%; specificity = 69.1%; Delta 7 am‐7 pm‐Lapses: AUC = 71%, 95% CI = [64–77], sensitivity = 50.9%; specificity = 83.3%).

Comparing IH patients with severe (*n* = 73) and without (*n* = 20) sleep inertia, the threshold of ≥ 4 for 7 am‐Lapses discriminated sleep inertia with a sensitivity of 72.6% and a specificity of 55% (AUC = 73%, 95% CI = [62–85]), whereas the threshold of ≥ 4 for Delta 7 am‐7 pm‐Lapses showed a sensitivity of 56.2% and a specificity of 80.0% (AUC = 68%, 95% CI = [56–80]). The threshold of ≥ 4 for 7 am‐Lapses also discriminated severe sleep inertia in IH patients with long sleep time (*n* = 62) compared with OSD patients without sleep inertia, with a sensitivity of 77.4% and a specificity of 69.1% (AUC = 79%, 95% CI = [71–87]), whereas the threshold of ≥ 4 for Delta 7 am‐7 pm‐Lapses showed a sensitivity of 59.7% and a specificity of 83.3% (AUC = 71.5%, 95% CI = [63–80]).

Finally, comparing OSD patients with severe (*n* = 90) and without (*n* = 84) sleep inertia the threshold of ≥ 4 for 7 am‐Lapses identified sleep inertia with a sensitivity of 64.4% and a specificity of 69.1% (AUC = 73%, 95% CI = [67–86]), whereas the threshold of ≥ 4 for Delta 7 am‐7 pm‐Lapses showed a sensitivity of only 46.7% and a specificity of 83.3% (AUC = 68%, 95% CI = [61–76]).

### 3.2 Clinical and Polysomnographic Characteristics Associated With High Number of PVT Lapses at Awakening

Among the global population of 350 patients, 199 (56.9%) had a number of 7 am‐Lapses ≥ 4 including 64.1% of IH, 61.3% of NSH, 55.6% of NT1, 44% of OSAS and 42.5% of the group “others” (Table 2). In comparison to those with 7 am‐Lapses < 4, these patients were predominantly female, more likely to have IH than other sleep disorders, and scored higher on total and partial IHSS, ESS, BDI‐II and 7:00‐KSS. They also reported more severe sleep inertia and sleep drunkenness, more hypersomnolence‐related driving impairment, higher night‐time sleep needs and an earlier habitual bedtime. Results remained unchanged after adjustment for age, sex and diagnosis, except for the BDI‐II score which became nonsignificant, and WASO which became higher in patients with 7 am‐Lapses ≥ 4 (Table 2, model 2). Among the whole population, Delta 7 am‐7 pm Lapses ≥ 4 were found in 139 participants (39.7%) including 47.3% of IH, 41.3% of NSH, 31.5% of NT1, 30% of OSAS and 35% of the group “others.” Compared to those below this threshold, these patients had similar characteristics as those with number of 7 am‐Lapses ≥ 4, except for BDI‐II and ESS scores, hypersomnolence‐related driving impairment, and habitual bedtime becoming non‐significantly associated. In addition, the percentage of N3 during the last hour of PSG was increased in patients with Delta 7 am‐7 pm‐Lapses ≥ 4; however, this between‐group difference was no longer significant after adjustment (Table 2, model 3).

**TABLE 2:** Clinical and polysomnographic characteristics associated with a high number of Psychomotor Vigilance Test (PVT) lapses at awakening in the whole population (*n* = 350).

	7 am‐Lapses < 4 *N* = 151	7 am‐Lapses ≥ 4 *N* = 199	*p* ^*a*^	*p* ^b^	Delta 7 am‐7 pm‐Lapses < 4 *N* = 211	Delta 7 am‐7 pm‐Lapses ≥ 4 *N* = 139	*p* ^a^	*p* ^b^	*p* ^c^
Demographic and clinical variables									
Age (years)^d^	30.98 (±10.81)	30.58 (±11.12)	0.73	—	31.66 (±11.29)	29.38 (±10.37)	0.06	—	—
Sex, female^e^	85 (56.29)	142 (71.36)	0.004	—	126 (59.72)	101 (72.66)	0.01	—	—
BMI (kg/m^2^)^d^	23.94 (±4.56)	23.98 (±4.97)	0.94	0.40	24.22 (±5.04)	23.56 (±4.37)	0.21	0.67	0.65
Diagnosis^e^									
IH	47 (31.13)	84 (42.21)	0.005		69 (32.70)	62 (44.60)	0.01	—	—
Other sleep disorders	104 (68.87)	115 (57.79)			142 (67.30)	77 (55.40)			
Treatment status, Naïve^e^	119 (78.81)	140 (70.35)	0.08	0.13	161 (76.30)	98 (70.50)	0.23	0.30	0.31
ESS score^d^	13.41 (±5.56)	15.29 (±4.54)	0.0008	0.005	14.14 (±5.35)	14.98 (±4.63)	0.13	0.22	0.24
ESS score, > 10^e^	103 (68.67)	169 (86.22)	0.0001	0.001	155 (74.52)	117 (84.78)	0.02	0.07	0.08
Total IHSS score^d^	27.09 (±9.98)	32.26 (±6.83)	< 0.0001	< 0.0001	28.27 (±9.41)	32.70 (±6.69)	< 0.0001	0.0002	0.0002
Total IHSS score, ≥ 26^e^	79 (59.40)	161 (85.19)	< 0.0001	< 0.0001	122 (65.24)	118 (87.41)	< 0.0001	0.0002	0.0001
Partial IHSS score^d^	23.48 (±8.16)	27.19 (±5.73)	< 0.0001	0.0003	24.40 (± 7.78)	27.40 (± 5.51)	0.0002	0.003	0.003
Self‐reported sleep inertia^e^									
Absent	69 (45.70)	35 (17.59)	< 0.0001	0.0001	86 (40.76)	18 (12.95)	< 0.0001	< 0.0001	< 0.0001
Mild	30 (19.87)	53 (26.63)			45 (21.33)	38 (27.34)			
Severe without drunkenness	36 (23.84)	63 (31.66)			49 (23.22)	50 (35.97)			
Severe with drunkenness	16 (10.60)	48 (24.12)			31 (14.69)	33 (23.74)			
Driving impairment, Moderate to very significant^e^	52 (34.44)	95 (47.74)	0.01	0.03	82 (38.86)	65 (46.76)	0.14	0.17	0.18
Night‐time sleep needs, ≥ 9 h^e^	73 (55.30)	136 (73.51)	0.0008	0.006	108 (58.06)	101 (77.10)	0.0005	0.01	0.01
Habitual bedtime (hh:mm)^d^	23:11 (± 1:00)	22:51 (± 1:04)	0.01	0.02	23:03 (±1:01)	22:53 (±1:06)	0.19	0.22	0.18
Habitual wake up time (hh:mm)^d^	7:50 (±1:39)	8:11 (±1:56)	0.14	0.13	7:56 (±1:47)	8:12 (±1:54)	0.24	0.37	0.36
BDI‐II score^d^	12.59 (±8.95)	14.95 (±9.93)	0.04	0.07	13.61 (±9.36)	14.52 (±9.94)	0.42	0.53	0.53
BDI‐II score^e^									
< 14	68 (57.14)	89 (50.57)	0.42	0.43	91 (53.22)	66 (53.23)	0.94	0.88	0.88
14–20	19 (15.97)	38 (21.59)			32 (18.71)	25 (20.16)			
≥ 20	32 (26.89)	49 (27.84)			48 (28.07)	33 (26.61)			
7:00‐KSS score^d^	5.73 (±2.03)	6.73 (±1.96)	< 0.0001	0.0002	5.89 (±2.01)	6.92 (±1.97)	< 0.0001	< 0.0001	< 0.0001
Polysomnography									
Sleep duration (min)^a^	425.51 (±58.07)	425.53 (±53.79)	0.99	0.34	424.20 (±55.70)	427.55 (±55.61)	0.58	0.53	0.54
Sleep efficiency (%)	84.46 (±10.80)	84.61 (±9.57)	0.89	0.32	84.11 (±10.52)	85.20 (±9.45)	0.39	0.75	0.77
Sleep latency (min)^a^	18.28 (±28.22)	20.53 (±56.28)	0.66	0.71	17.23 (±25.03)	23.09 (±66.63)	0.31	0.34	0.33
REM sleep latency (min)^d^	90.88 (±57.71)	86.46 (±58.14)	0.48	0.22	91.40 (±62.39)	83.75 (±50.28)	0.23	0.14	0.14
WASO (min)^d^	52.93 (±40.74)	56.65 (±46.43)	0.44	0.04	56.94 (±45.47)	52.17 (±41.78)	0.32	0.74	0.76
N1 (%)^a^	5.50 (±4.16)	5.63 (±5.24)	0.80	0.18	5.57 (±4.76)	5.58 (±4.88)	0.99	0.20	0.21
N2 (%)^a^	54.20 (±8.05)	53.29 (±8.57)	0.31	0.25	53.57 (±8.61)	53.84 (±7.97)	0.77	0.80	0.80
N3 (%)^a^	19.07 (±6.24)	19.41 (±6.47)	0.62	0.86	19.16 (±6.11)	19.42 (±6.76)	0.71	0.67	0.68
REM (%)^a^	21.18 (±5.93)	21.62 (±5.93)	0.50	0.45	21.64 (±6.21)	21.11 (±5.48)	0.41	0.40	0.39
PLMS index (/hour)^d^	6.70 (±12.44)	8.48 (±17.75)	0.30	0.07	7.90 (±15.16)	7.42 (±16.51)	0.78	0.47	0.47
AHI (/hour)^a^	5.84 (±11.02)	4.20 (±7.48)	0.11	0.76	5.80 (±10.82)	3.55 (±5.70)	0.03	0.42	0.42
Micro‐arousal index (/hour)^d^	13.66 (±10.29)	12.71 (±7.62)	0.32	0.91	14.00 (±9.82)	11.77 (±7.03)	0.02	0.22	0.21
Last hour N1 (%)^a^	5.35 (±5.66)	5.34 (±5.52)	0.99	0.69	5.41 (±5.95)	5.25 (±4.99)	0.79	0.93	0.41
Last hour N2 (%)^a^	48.33 (±23.93)	44.26 (±24.59)	0.13	0.10	46.96 (±24.79)	44.56 (±23.71)	0.37	0.32	0.30
Last hour N3 (%)^d^	3.32 (±9.67)	4.46 (±11.35)	0.33	0.33	2.99 (±8.82)	5.45 (±12.83)	0.04	0.05	0.72
Last hour REM (%)^d^	28.91 (±21.22)	29.07 (±22.17)	0.95	0.88	16.76 (±12.76)	18.36 (±13.44)	0.27	0.39	0.74
Last hour WAKE (%)^d^	14.35 (±21.79)	17.16 (±25.33)	0.28	0.19	17.01 (±25.47)	14.40 (±21.31)	0.32	0.46	0.70
MSLT (*N* = 307)									
MSL on MSLT (min)^a^	9.72 (±4.57)	10.28 (±5.12)	0.33	0.34	9.69 (±4.88)	10.57 (±4.91)	0.12	0.08	0.08
Total SOREMPS on MSLT/PSG^d^	0.82 (±1.71)	0.86 (±1.81)	0.86	0.15	0.93 (±1.84)	0.73 (±1.66)	0.34	0.64	0.64

Abbreviations: AHI = apnea‐hypopnea index, BDI‐II = Beck Depression Inventory II, BMI = body mass index, ESS = Epworth Sleepiness Scale, IHSS = Idiopathic Hypersomnia Severity Scale, KSS = Karolinska Sleepiness Scale, MSL = mean sleep latency, MSLT = multiple sleep latency test, N1 = sleep stage N1, N2 = sleep stage N2, N3 = sleep stage N3, PLMS = periodic limb movements during sleep, REM = rapid eye movement, WASO = wake after sleep onset.

^a^ Crude association.

^b^ Adjustment for sex, age and diagnosis.

^c^ Adjustment for sex, age, diagnosis and the number of PVT lapses at 7:00 p.m.

^d^ Quantitative variables are expressed as means (±standard deviation).

^e^ Categorical variables are shown as *n* (%).

Among all patients with IH, 84 (64.1%) had a 7 am‐Lapse number ≥ 4 (Table 3). Compared to those with IH and 7 am‐Lapses < 4, these patients reported more frequently severe sleep inertia associated with sleep drunkenness and exhibited higher 7:00‐KSS scores. No differences were found for age, sex, or PSG variables. Similar associations were observed when comparing patients with IH who had a Delta 7 am‐7 pm‐Lapses ≥ 4 (*n* = 62, 47.3%) with those below this threshold, even after adjusting for the number of PVT lapses at 7:00 pm (Table 3, Model 2). Among the 106 patients with IH with long sleep time, those with 7 am‐Lapses ≥ 4 reported more frequently severe sleep inertia than those with 7 am‐Lapses < 4 (*p* = 0.02), with the same trend for Delta 7 am‐7 pm‐Lapses ≥ 4 (*p* = 0.09); no difference was found for patients with IH without long sleep time (data not shown).

**TABLE 3:** Clinical and polysomnographic characteristics associated with a high number of Psychomotor Vigilance Test (PVT) lapses at awakening in patients with idiopathic hypersomnia (*N* = 131).

	7 am‐Lapses < 4 *N* = 47	7 am‐Lapses ≥ 4 *N* = 84	*p*	Delta 7 am‐7 pm‐Lapses < 4 *N* = 69	Delta 7 am‐7 pm‐Lapses ≥ 4 *N* = 62	*p* ^a^	*p* ^b^
Demographic and clinical variables							
Age (years)^c^	26.90 (±7.04)	28.23 (±9.94)	0.42	28.83 (±9.58)	26.55 (±8.22)	0.15	0.21
Sex, female^d^	37 (78.72)	71 (84.52)	0.40	55 (79.71)	53 (85.48)	0.39	0.34
BMI (kg/m^2^)^a^	22.46 (±3.88)	22.91 (±4.41)	0.55	22.63 (±4.20)	22.88 (±4.27)	0.74	0.73
Treatment status, Naïve^d^	33 (70.21)	57 (67.86)	0.76	48 (69.57)	42 (67.74)	0.82	0.78
ESS score ^c^	14.74 (±4.33)	15.77 (±4.05)	0.18	15.35 (±4.35)	15.47 (±3.99)	0.87	0.74
ESS score, > 10^d^	39 (84.78)	76 (90.48)	0.34	59 (86.76)	56 (90.32)	0.53	0.42
Total IHSS score ^c^	31.44 (±6.79)	34.04 (±7.55)	0.07	31.83 (±7.26)	34.50 (±7.30)	0.046	0.03
Total IHSS score, ≥ 26^d^	34 (79.07)	72 (90.00)	0.10	51 (80.95)	55 (91.67)	0.09	0.07
Partial IHSS score^c^	27.00 (±5.01)	28.48 (±6.06)	0.18	27.29 (±5.67)	28.67 (±5.77)	0.18	0.13
Self‐reported sleep inertia^d^							
Absent	11 (23.40)	9 (10.71)	0.03	16 (23.19)	4 (6.45)	0.05	0.04
Mild	16 (34.04)	22 (26.19)		21 (30.43)	17 (27.42)		
Severe without drunkenness	14 (29.79)	23 (27.38)		17 (24.64)	20 (32.26)		
Severe with drunkenness	6 (12.77)	30 (35.71)		15 (21.74)	21 (33.87)		
Driving impairment, Moderate to very significant^d^	23 (48.94)	46 (54.76)	0.52	36 (52.17)	33 (53.23)	0.90	0.88
Night‐time sleep needs, ≥ 9 h^d^	33 (76.74)	67 (84.81)	0.27	48 (76.19)	52 (88.14)	0.09	0.07
Habitual bedtime (hh:mm)^c^	23:10 (±1:03)	22:56 (±0:59)	0.21	22:59 (±1:04)	23:03 (±0:58)	0.71	0.91
Habitual wake up time (hh:mm)^c^	8:01 (±1:46)	8:19 (±1:58)	0.38	8:06 (±1:50)	8:19 (±1:59)	0.52	0.54
BDI‐II score^c^	12.07 (±8.65)	15.05 (±10.87)	0.12	12.93 (±9.52)	15.18 (±10.88)	0.21	0.22
BDI‐II score^d^							
< 14	26 (57.78)	44 (53.01)	0.85	37 (55.22)	33 (54.10)	0.99	0.99
14–20	8 (17.78)	15 (18.07)		12 (17.91)	11 (18.03)		
≥ 20	11 (24.44)	24 (28.92)		18 (26.87)	17 (27.87)		
7:00‐KSS score^c^	6.32 (±2.12)	7.24 (±1.82)	0.01	6.37 (±2.10)	7.48 (±1.67)	0.002	0.002
Polysomnography							
Sleep duration (min)^a^	449.97 (±41.19)	446.40 (±37.82)	0.62	447.51 (±40.72)	447.92 (±37.18)	0.95	0.94
Sleep efficiency (%)^c^	89.77 (±5.22)	88.64 (±6.09)	0.28	89.12 (±5.72)	88.96 (±5.93)	0.88	0.84
Sleep latency (min)^a^	14.12 (±14.32)	22.78 (±83.68)	0.57	13.20 (±12.59)	26.87 (±97.17)	0.49	0.48
REM sleep latency (min)^c^	98.17 (±54.07)	95.29 (±48.09)	0.75	101.57 (±57.47)	90.48 (±40.10)	0.21	0.21
WASO (min)^c^	32.59 (±22.45)	39.55 (±29.84)	0.17	36.72 (±27.44)	37.41 (±27.85)	0.89	0.83
N1 (%)^a^	3.94 (±2.51)	4.13 (±3.00)	0.71	3.92 (±2.64)	4.21 (±3.04)	0.56	0.54
N2 (%)^a^	54.77 (±6.91)	54.09 (±7.39)	0.60	54.86 (±6.73)	53.74 (±7.71)	0.37	0.35
N3 (%)^a^	19.59 (±5.03)	19.90 (±6.73)	0.78	19.44 (±5.42)	20.19 (± 6.91)	0.49	0.51
REM (%)^a^	21.64 (±4.61)	21.85 (±5.05)	0.82	21.73 (±4.30)	21.82 (± 5.49)	0.91	0.83
PLMS index (/hour)^c^	5.07 (±8.71)	4.77 (±8.74)	0.85	5.08 (±8.05)	4.66 (±9.43)	0.78	0.79
AHI (/hour)^a^	2.85 (±3.62)	2.08 (±2.58)	0.17	2.60 (±3.31)	2.09 (±2.63)	0.33	0.32
Micro‐arousal index (/hour)^c^	10.56 (±7.25)	10.04 (±5.09)	0.63	10.77 (±6.69)	9.62 (±4.93)	0.27	0.30
Last hour N1 (%)^a^	4.20 (±4.50)	4.49 (±4.62)	0.73	4.43 (±4.56)	4.33 (±4.59)	0.90	0.67
Last hour N2 (%)^a^	49.24 (±23.28)	45.67 (±23.81)	0.41	47.20 (±24.11)	46.69 (±23.19)	0.90	0.46
Last hour N3 (%)^c^	4.15 (±10.64)	4.98 (±13.60)	0.72	3.02 (±8.97)	6.56 (±15.55)	0.12	0.70
Last hour REM (%)^c^	32.20 (±21.64)	30.17 (±21.72)	0.61	31.45 (±22.46)	30.29 (±20.82)	0.76	0.93
Last hour WAKE (%)^c^	10.50 (±15.76)	14.93 (±23.75)	0.26	14.21 (±23.12)	12.32 (±19.05)	0.61	0.51
MSLT (*N* = 131)							
MSL on MSLT (min)^a^	7.64 (±3.39)	8.88 (±4.07)	0.08	7.84 (±3.56)	9.09 (±4.12)	0.07	0.05
MSL on MSLT, ≤ 8 min^d^	28 (59.57)	39 (46.43)	0.15	38 (55.07)	29 (46.77)	0.34	0.33
Total SOREMPS on MSLT/PSG^c^	0.13 (±0.40)	0.13 (±0.53)	0.97	0.10 (±0.35)	0.16 (±0.61)	0.49	0.54
Bed rest (*N* = 121)							
32‐h bed‐rest TST (hour)^c^	21.30 (±3.13)	21.05 (±2.57)	0.64	20.93 (±3.21)	21.36 (±2.21)	0.40	0.46
32‐h bed‐rest TST, ≥ 19 h^d^	35 (83.33)	71 (89.87)	0.30	51 (82.26)	55 (93.22)	0.08	0.08

Abbreviations: AHI = apnea‐hypopnea index, BDI‐II = Beck Depression Inventory II, BMI = body mass index, ESS = Epworth Sleepiness Scale, IHSS = Idiopathic Hypersomnia Severity Scale, KSS = Karolinska Sleepiness Scale, MSL = mean sleep latency, MSLT = multiple sleep latency test, N1 = sleep stage N1, N2 = sleep stage N2, N3 = sleep stage N3, PLMS = periodic limb movements during sleep, REM = rapid eye movement, TST = total sleep time, WASO = wake after sleep onset.

^a^ Crude association.

^b^ Adjustment for the number of PVT lapses at 7:00 p.m.

^c^ Quantitative variables are expressed as means (± standard deviation).

^d^ Categorical variables are shown as *n* (%).

Among all other OSD patients, 115 (52.5%) had 7 am‐Lapses ≥ 4 (Table 4). Compared to those with 7 am‐Lapses < 4, these patients were more frequently female, had higher total and partial IHSS, ESS, and 7:00‐KSS scores, reported more severe sleep inertia and sleep drunkenness, more hypersomnolence‐related driving impairment, higher night‐time sleep needs, and earlier habitual bedtime. Results remained unchanged after adjustment for sex and diagnosis, except for hypersomnolence‐related driving impairment, becoming borderline significant (*p* = 0.05) (Table 4, Model 2). A total of 77 (35.2%) OSD patients had a Delta 7 am‐7 pm‐Lapse number ≥ 4. Compared to those with Delta 7 am‐7 pm‐Lapses < 4, they had higher total and partial IHSS and 7:00‐KSS scores, reported more severe sleep inertia and sleep drunkenness, higher night‐time sleep needs, and an earlier habitual bedtime. Furthermore, a lower percentage of N2 was observed during the last hour of the PSG after adjusting for sex, diagnosis, and the number of PVT lapses at 7:00 pm (Table 4, Model 3).

**TABLE 4:** Clinical and polysomnographic characteristics associated with a high number of Psychomotor Vigilance Test (PVT) lapses at awakening in patients with other sleep disorders (*N* = 219).

	7 am‐Lapses < 4 *N* = 104	7 am‐Lapses ≥ 4 *N* = 115	*p* ^a^	*p* ^b^	Delta 7 am‐7 pm‐Lapses < 4 *N* = 142	Delta 7 am‐7 pm‐Lapses ≥ 4 *N* = 77	*p* ^a^	*p* ^b^	*p* ^c^
Demographic and clinical variables									
Age (years)^d^	32.82 (±11.70)	32.30 (±11.65)	0.74	0.59	33.04 (±11.82)	31.65 (±11.36)	0.40	0.61	0.52
Sex, female^e^	56 (53.85)	44 (38.26)	0.02	—	71 (50.00)	48 (62.34)	0.08	—	—
BMI (kg/m^2^)^d^	24.61 (±4.70)	24.76 (±5.23)	0.82	0.57	25.00 (±5.25)	24.12 (±4.40)	0.21	0.37	0.29
Treatment status, Naïve^e^	86 (82.69)	83 (72.17)	0.07	0.09	113 (79.58)	56 (72.73)	0.25	0.19	0.20
Diagnosis^e^									
NSH	29 (27.88)	46 (40.00)	0.14	—	44 (30.99)	31 (40.26)	0.54	—	—
NT1	24 (23.08)	30 (26.09)			37 (26.06)	17 (22.08)			
OSAS	28 (26.92)	22 (19.13)			35 (24.65)	15 (19.48)			
Other	23 (22.12)	17 (14.78)			26 (18.31)	14 (18.18)			
ESS score^d^	12.82 (±5.94)	14.93 (±4.87)	0.005	0.03	13.55 (±5.70)	14.58 (±5.08)	0.19	0.15	0.21
ESS score, > 10^e^	64 (61.54)	93 (83.04)	0.0005	0.004	96 (68.57)	61 (80.26)	0.07	0.05	0.07
Total IHSS score^d^	25.01 (±10.61)	30.96 (±5.96)	< 0.0001	0.0002	27.00 (5.00; 44.00)	31.00 (19.00; 42.00)	0.0003	0.0009	0.001
Total IHSS score, ≥ 26^e^	45 (50.00)	89 (81.65)	< 0.0001	< 0.0001	71 (57.26)	63 (84.00)	0.0002	0.0004	0.0005
Partial IHSS score^d^	21.80 (±8.84)	26.23 (±5.30)	< 0.0001	0.001	22.93 (±8.30)	26.38 (±5.11)	0.002	0.006	0.006
Self‐reported sleep inertia^e^									
Absent	58 (55.77)	26 (22.61)	< 0.0001	< 0.001	70 (49.30)	14 (18.18)	< 0.001	0.0007	0.001
Mild	14 (13.46)	31 (26.96)			24 (16.90)	21 (27.27)			
Severe without drunkenness	22 (21.15)	40 (34.78)			32 (22.54)	30 (38.96)			
Severe with drunkenness	10 (9.62)	18 (15.65)			16 (11.27)	12 (15.58)			
Driving impairment, Moderate to very significant^e^	29 (27.88)	49 (42.61)	0.02	0.05	46 (32.39)	32 (41.56)	0.18	0.16	0.20
Night‐time sleep needs, ≥ 9 h^e^	40 (44.94)	69 (65.09)	0.005	0.03	60 (48.78)	49 (68.06)	0.009	0.03	0.02
Habitual bedtime (hh:mm)^d^	23:12 (±0:58)	22:45 (±1:09)	0.02	0.04	23:06 (±0:59)	22:42 (±1:12)	0.03	0.03	0.03
Habitual wake up time (hh:mm)^d^	7:43 (±1:34)	8:03 (±1:55)	0.28	0.26	7:47 (±1:45)	8:04 (±1:48)	0.36	0.46	0.46
BDI‐II score^d^	12.91 (±9.17)	14.86 (±9.08)	0.17	0.37	14.06 (±9.28)	13.89 (±8.98)	0.91	0.73	0.70
BDI‐II score^e^									
< 14	42 (56.76)	45 (48.39)	0.28	0.41	54 (51.92)	33 (52.38)	0.84	0.70	0.71
14–20	11 (14.86)	23 (24.73)			20 (19.23)	14 (22.22)			
≥ 20	21 (28.38)	25 (26.88)			30 (28.85)	16 (25.40)			
7:00‐KSS score^d^	5.47 (±1.95)	6.38 (±1.99)	0.001	0.001	5.67 (±1.93)	6.47 (±2.08)	0.007	0.01	0.01
Polysomnography									
Sleep duration (min)^a^	414.45 (±61.29)	410.52 (±58.51)	0.63	0.15	412.79 (±58.55)	411.68 (±62.27)	0.89	0.70	0.78
Sleep efficiency (%)^d^	82.05 (±11.79)	81.67 (±10.56)	0.80	0.33	81.68 (±11.44)	82.17 (±10.63)	0.76	0.98	0.86
Sleep latency (min)^a^	20.16 (±32.51)	18.88 (±19.92)	0.72	0.99	19.19 (±29.07)	20.04 (±21.45)	0.82	0.89	0.92
REM sleep latency (min)^d^	87.55 (±59.26)	80.00 (±63.94)	0.37	0.47	86.42 (±64.27)	78.34 (±56.87)	0.36	0.16	0.16
WASO (min)^d^	62.13 (±43.79)	69.14 (±52.19)	0.29	0.11	66.76 (±49.19)	64.06 (±47.18)	0.69	0.99	0.90
N1 (%)^a^	6.21 (±4.55)	6.73 (±6.19)	0.48	0.25	6.37 (±5.33)	6.68 (±5.74)	0.69	0.36	0.40
N2 (%)^a^	53.94 (±8.54)	52.70 (±9.32)	0.31	0.44	52.95 (±9.35)	53.93 (±8.23)	0.44	0.45	0.45
N3 (%)^a^	18.84 (±6.73)	19.06 (±6.27)	0.80	0.71	19.03 (±6.42)	18.80 (±6.62)	0.80	0.42	0.46
REM (%)^a^	20.97 (±6.45)	21.45 (±6.52)	0.59	0.68	21.60 (±6.96)	20.53 (±5.44)	0.24	0.33	0.32
PLMS index (/hour)^d^	7.44 (± 13.78)	11.19 (±21.78)	0.14	0.08	9.28 (±17.47)	9.64 (±20.30)	0.89	0.48	0.51
AHI (/hour)^a^	7.20 (±12.84)	5.75 (±9.31)	0.34	0.89	7.36 (±12.71)	4.72 (±7.09)	0.11	0.30	0.30
Micro‐arousal index (/hour)^d^	15.05 (±11.16)	14.65 (±8.54)	0.76	0.80	15.57 (±10.69)	13.50 (±7.96)	0.14	0.27	0.24
Last hour N1 (%)^a^	5.89 (±6.08)	5.96 (±6.04)	0.93	0.92	5.91 (±6.49)	5.97 (±5.20)	0.64	0.80	0.80
Last hour N2 (%)^a^	47.90 (±24.33)	43.22 (±25.19)	0.17	0.22	46.84 (±25.21)	42.87 (±24.13)	0.13	0.02	0.03
Last hour N3 (%)^d^	2.93 (±9.21)	4.08 (±9.43)	0.37	0.36	2.97 (±8.78)	4.57 (±10.20)	0.43	0.29	0.35
Last hour REM (%)^d^	27.37 (±20.95)	28.27 (±22.55)	0.76	0.84	26.17 (±20.49)	30.83 (±23.72)	0.30	0.27	0.32
Last hour WAKE (%)^d^	16.17 (±23.97)	18.78 (±26.41)	0.45	0.47	18.41 (±26.55)	16.04 (±22.93)	0.60	0.33	0.34
MSLT (*N* = 176)									
MSL on MSLT (min)^a^	10.99 (±4.74)	11.48 (±5.62)	0.37	0.31	10.85 (±5.24)	11.94 (±5.21)	0.18	0.54	0.61
Total SOREMPS on MSLT/PSG^d^	1.25 (±2.03)	1.47 (±2.23)	0.49	0.09	1.45 (±2.18)	1.25 (±2.10)	0.55	0.29	0.48

Abbreviations: AHI = apnea‐hypopnea index, BDI‐II = Beck Depression Inventory II, BMI = body mass index, ESS = Epworth Sleepiness Scale, IHSS = Idiopathic Hypersomnia Severity Scale, KSS = Karolinska Sleepiness Scale, MSL = mean sleep latency, MSLT = multiple sleep latency test, N1 = sleep stage N1, N2 = sleep stage N2, N3 = sleep stage N3, PLMS = periodic limb movements during sleep, REM = rapid eye movement, WASO = wake after sleep onset.

^a^ Crude association.

^b^ Adjustment for sex and diagnosis.

^c^ Adjustment for sex, diagnosis and the number of PVT lapses at 7:00 p.m.

^d^ Quantitative variables are expressed as means (±standard deviation).

^e^ Categorical variables are shown as *n* (%).

### 3.3 Additional PVT Metrics to Objectively Measure Severe Sleep Inertia in IH Patients Compared With Those With OSD Patients Without Sleep Inertia

Comparison of additional PVT metrics (mean and median RT, 1/RT, slowest 10% 1/RT, fastest 10% RT, and lapses + false starts) with the lapse‐based outcomes showed generally small and non‐significant differences in AUC except for the slowest 10% 1/RT with lower AUC (Table S2). Similarly, no delta metric improved discriminative performance compared with lapse‐based measures (Table S3).

Given the stronger AUC and narrower confidence interval observed for mean 1/RT metric, optimal thresholds were determined for 7 am‐mean 1/RT and delta 7 am–7 pm‐mean 1/RT to identify sleep inertia by comparing IH patients with severe sleep inertia (*n* = 73) and OSD patients without sleep inertia (*n* = 84). The optimal thresholds were: 7 am‐mean 1/RT < 2.60 (AUC = 78%, 95% CI = [71–86]; sensitivity = 66.7%, 95% CI = [54–77]; specificity = 79.8%, 95% CI = [66–88]), and delta 7 am–7 pm mean 1/RT < –0.26 (AUC = 76, 95% CI = [68–84]; sensitivity = 72.2%, 95% CI = [60–82]; specificity = 72.3%, 95% CI = [61–82]).

Applying these thresholds in the full cohort and in IH and OSD subgroups separately yielded similar clinical and neurophysiological associations to lapse‐based cutoffs (Table S4–S6). Additionally, IH patients below the 7 am‐mean 1/RT cutoff showed higher IHSS scores, while those below the delta 7 am–7 pm‐mean 1/RT cutoff were younger and had more often a TST ≥ 19 h/32. OSD patients below the 7 am‐mean 1/RT and the delta 7 am–7 pm‐mean 1/RT had more frequently a diagnosis of NSH.

## 4 Discussion

This study determined the optimal cut‐off values for the number of PVT lapses (i.e., RT ≥ 500 msec) at awakening after nighttime sleep to objectively identify sleep inertia by comparing IH patients who reported severe sleep inertia with patients with other sleep disorders who did not report sleep inertia. Thresholds of ≥ 4 for 7 am‐Lapses and ≥ 4 for Delta 7 am‐7 pm‐Lapses best identified reduced performance at awakening associated with severe sleep inertia in IH, and also showed adequate performance in other sleep disorders. In the full cohort (IH and OSD), 56.9% of patients had ≥ 4 7 am‐Lapses, and 39.7% had ≥ 4 Delta 7 am‐7 pm Lapses. A number of lapses ≥ 4 was associated with female sex, higher ESS, IHSS and KSS scores, increased frequency of severe self‐reported sleep inertia and sleep drunkenness, more hypersomnolence‐related driving impairment, higher night‐time sleep needs and an earlier habitual bedtime. In IH patients, a number of lapses ≥ 4 was associated with severe self‐reported sleep inertia, sleep drunkenness, and higher KSS scores. Among the other PVT metrics evaluated, mean 1/RT also demonstrated good discriminative performance to objectively measure severe sleep inertia in IH patients compared with patients with other sleep disorders without sleep inertia.

In the context of sleep inertia in the general population, the magnitude of impaired performance upon awakening may be similar to that observed after 40 h of sleep deprivation (Rosa et al. 1983) and can be associated with severe real‐world consequences (Hilditch and McHill 2019; Horne and Moseley 2011). Patients with IH frequently report sleep inertia that is pronounced, long‐lasting, and associated with significant, debilitating impairment in daily functioning. (Trotti 2017; Ruby et al. 2024; Billiard and Dauvilliers 2001; Anderson et al. 2007). The limited number of studies that have investigated sleep inertia found that, compared to control subjects, patients with central disorders of hypersomnolence had reduced alertness upon awakening from nighttime sleep or after a nap, with delayed cognitive evoked potentials (P300) (Bastuji et al. 2003; Peter‐Derex et al. 2013) and a higher number of PVT lapses (Evangelista et al. 2022; Trotti et al. 2022). In this last study, PVT performance worsened following naps in patients with hypersomnolence, irrespective of the etiologies; however, no self‐assessment of sleep inertia was included (Trotti et al. 2022). We previously reported that patients with severe sleep inertia as assessed by the IHSS had a marked increase of PVT lapses at awakening (Evangelista et al. 2022); however, both sleep inertia and PVT lapses varied considerably among patients with central hypersomnolence disorders. Therefore, defining an optimal PVT cut‐off is important to identify patients at risk of severe performance impairment upon awakening and to provide an objective marker of sleep inertia, a phenomenon that remains difficult to quantify and is typically assessed subjectively. However, our aim was not to replace self‐reported measures, but to complement them by proposing an objective marker of the functional impact of severe sleep inertia on sustained attention. There is a need for objective criteria to assess sleep inertia, similar to the use of MSLT latency for quantifying daytime sleepiness and extended sleep recordings for quantifying hypersomnia.

Here, we report that PVT thresholds of ≥ 4 for 7 am‐Lapses (sensitivity = 72.6%, specificity = 69.1%) along with an increase of ≥ 4 lapses from 07:00 pm to 07:00 am (sensitivity = 50.9%, specificity = 83.3%) appear to best capture performance impairment related to severe sleep inertia in IH compared with OSD without sleep inertia. Alternative PVT metrics (mean and median RT, 1/RT, slowest 10% 1/RT, fastest 10% RT, and lapses + false starts) did not improve discriminative accuracy and were less clinically intuitive. Also, patients with 7 am‐Lapses ≥ 4 had an increased impairment in their driving performances, increased levels of sleep inertia and sleep drunkenness, and a lower level of alertness as assessed by the KSS. Most of these patients were diagnosed with IH (64.1% and 47.3% for 7 am‐Lapses ≥ 4 and Delta 7 am‐7 pm Lapses ≥ 4, respectively) and especially in its form with long sleep time (67.0% and 51.9% for 7 am‐Lapses ≥ 4 and Delta 7 am‐7 pm Lapses ≥ 4, respectively). The mean 1/RT measure also effectively discriminates severe sleep inertia in IH compared with OSD patients without sleep inertia.

Although a pronounced sleep inertia is typical and frequent in IH, it is not exclusive to this condition (Evangelista et al. 2022). We found that a large proportion of patients with other sleep disorders also had 7 am‐Lapses ≥ 4 and Delta 7 am‐7 pm‐Lapses ≥ 4, ranging from 42.5% to 61.3% and 30% to 41.3%, respectively. The group designated as “non‐specified hypersomnolence” comprised 61.3% and 41.3% of patients exhibiting 7 am‐Lapses ≥ 4 and Delta 7 am‐7 pm Lapses ≥ 4, respectively. This heterogeneous condition presents clinical symptoms similar to those observed in IH but lacks the objective ICSD‐3 TR criteria for this disease (American Academy of Sleep Medicine 2023; Evangelista et al. 2018). Sleep is classically considered refreshing in NT1, but some patients reported difficulty waking up and even a severe sleep inertia (Evangelista et al. 2022; Bassetti et al. 2003; Martínez‐Rodríguez et al. 2007). We found here that 55.6% of patients with NT1 had 7 am‐Lapses ≥ 4 and 31.5% had Delta 7 am‐7 pm‐Lapses ≥ 4, results consistent with previous studies using different paradigms to document decreased performance upon awakening (Peter‐Derex et al. 2013; Mullington and Broughton 1994; Godbout and Montplaisir 1986). We also reported that patients with OSAS (44% and 30% for 7 am‐Lapses ≥ 4 and Delta 7 am‐7 pm‐Lapses ≥ 4, respectively), insomnia, and restless leg syndrome (42.5% and 35% for 7 am‐Lapses ≥ 4 and Delta 7 am‐7 pm‐Lapses ≥ 4, respectively) had also poor performance upon awakening potentially linked to chronic sleep deprivation/fragmentation (Ohayon et al. 2000; Chugh et al. 1996).

Little is known about the underlying mechanisms causing severe sleep inertia, with conflicting findings in the literature (Trotti 2017; Ruby et al. 2024; Hilditch and McHill 2019). Female sex was associated with a 7 am‐Lapses ≥ 4 and a Delta 7 am‐7 pm‐Lapses ≥ 4 in patients with different sleep disorders, whereas no such association was found in IH patients. However, the relationship between gender, sleep inertia, and performance upon awakening, and whether this relationship varies according to the underlying sleep disorder, remains unclear. A recent study indicated that, although women reported feeling sleepier than men following nocturnal awakenings, their cognitive performance was comparable (Hilditch et al. 2023). Sleep inertia is more frequently reported in young individuals (Ohayon et al. 2000; Ohayon et al. 2014). In our study, younger age was associated with reduced psychomotor speed at awakening compared to 07:00 pm (Delta 7 am‐7 pm‐mean 1/RT), but not with the number of lapses. A restricted age range, with a relatively young cohort (mean age < 30 years), may have limited the ability to detect age‐related effects on PVT performance. Performance impairment at awakening was associated with self‐reported EDS (i.e., ESS score > 10 and higher IHSS, excluding or not the three items related to sleep inertia). However, no significant association was identified between PVT performance and objective EDS on the MSLT, thereby confirming the discrepancy between self‐report and objective measures (Evangelista et al. 2021).

Sleep inertia likely interacts with the homeostatic process to influence performance immediately after awakening (Trotti 2017; Ruby et al. 2024; Hilditch and McHill 2019). However, no association was identified between PVT performance and sleep stages, particularly slow wave sleep, or self‐reported sleep needs in patients with IH. Also, objective sleep duration measured by PSG was only weakly associated with the mean 1/RT metric (and not with lapses). A dysfunction in sleep homeostasis has been proposed in IH (Adam et al. 2026; Pizza et al. 2013; Sforza et al. 2000), where patients frequently experience a prolonged unmet need for sleep, potentially leading to increased sleep pressure and exacerbated sleep inertia. Accordingly, slow wave sleep close to awakening (after 06:00 am) has been reported to be higher in IH patients than controls (Vernet and Arnulf 2009); however, we found no such differences between IH patients with and without severe sleep inertia. It remains unclear whether lapses at awakening reflect a weak arousal drive due to an instability of the arousal system to restore a complete and rapid awakening or to an excessive sleep drive with microsleeps induced by higher sleep pressure (Miccoli et al. 2008). Of interest, sodium oxybate and lower‐sodium oxybate reduce EDS, prolong nighttime sleep, and improve sleep inertia in IH, although their mechanisms remain unclear (Leu‐Semenescu et al. 2016; Dauvilliers et al. 2022). Assessing the impact of these medications on PVT outcomes would provide important insights.

Circadian processes may also influence sleep inertia with stronger effects when awakening occurs near the circadian temperature trough, as observed in delayed sleep phase syndrome (Trotti 2017; Ruby et al. 2024; Hilditch and McHill 2019). Circadian alterations may also contribute to the symptoms of IH (Landzberg and Trotti 2019) and lower scores on the Horne and Ostberg questionnaire (indicating an evening type) have been observed in some patients with IH and sleep inertia (Vernet et al. 2010). However, a recent study using core body temperature found no difference in circadian phase or amplitude between patients with IH and other populations (Adam et al. 2025). Furthermore, we did not observe an evening chronotype in patients with impaired PVT performance at awakening; instead, impaired PVT performance was associated with an earlier habitual bedtime.

Sleep inertia has been frequently reported in patients with mood disorders (Ohayon et al. 2000; Ohayon et al. 2014; Kanady and Harvey 2015) and has been associated with depressive symptoms in patients with sleep disorders (Evangelista et al. 2022). The present study found a weak association between PVT performance upon awakening and depressive symptoms as assessed by BDI‐II in the whole population, although this association did not persist after multiple adjustments. Similarly, patients with both hypersomnolence and psychiatric disorders showed event‐related potential responses similar to controls (Bastuji et al. 2003; Peter‐Derex et al. 2013). Further investigations are needed to clarify whether sleep inertia in depression is more related to anhedonia or decreased motivation than to real altered sleep–wake transition.

This study has several limitations. Although PVT thresholds (7 am‐Lapses ≥ 4 and Delta 7 am‐7 pm Lapses ≥ 4) represent the optimal thresholds to identify severe sleep inertia in IH compared with OSD without sleep inertia, their sensitivity and specificity remain moderate. Other neurobehavioral tests, such as the sustained attention to response task (Fronczek et al. 2006), may provide additional information. Our study was performed in the context of a sleep disorder diagnostic evaluation, explaining the lack of healthy controls and the heterogeneity of sleep disorders. We were also underpowered to study properly sleep inertia according to the different IH phenotypes, with only 12.4% of patients having the form without long sleep time. Circadian assessment was limited to habitual bedtime and wake‐up time without objective markers such as actigraphy or dim light melatonin onset. Participants were allowed to sleep from 11:00 pm until 7:00 am in the sleep unit, which may be different from their home habits. Such standardized procedure in the sleep unit may result in a reduced amount of sleep compared to patients' sleep needs, which may have an impact on the results. This is particularly the case in patients with IH, who often require more than 9 h of nocturnal sleep, and are forced to wake up at 07:00 am accordingly. We found no association between sleep stage distribution during the last hour before the PVT and PVT lapses; however, PVT performance was not assessed according to the sleep stage immediately preceding the 07:00 am PVT. Sleep inertia was assessed in a standardized manner over the last month using two IHSS items evaluating its frequency and duration. The presence of sleep drunkenness, a behavioural component of sleep inertia related to its severity, was analysed here only in the presence of severe sleep inertia. The 5‐min PVT was used to enable repeated measurements while limiting weariness and lack of motivation in participants. Further studies should evaluate sleep inertia in real‐life settings using portable devices and repeated measures, and consider additional factors such as motivation to wake up. Finally, the generalizability of PVT cutoffs may be limited by differences in awakening times, sleep duration, and comparator group characteristics (e.g., variations in age or the distribution of sleep disorders).

## 5 Conclusion

The results demonstrated that a PVT lapse threshold of ≥ 4 upon awakening (07:00 am), as well as a difference of ≥ 4 lapses relative to the evening reference time (07:00 pm the previous day), represent optimal cut‐offs for objectively capturing sleep inertia–related performance impairment in patients with idiopathic hypersomnia, while also showing adequate performance in patients with other sleep disorders. The threshold of 4 PVT lapses at awakening demonstrated the best balance between sensitivity and specificity and, as it relies on a single 5‐min PVT administration, is practical and well‐suited to clinical settings, clinical trials, and other research protocols to optimize the management of this disabling symptom.

## Author Contributions

**Elisa Evangelista:** writing – original draft, investigation, methodology. **Alexandre Derré:** investigation, writing – review and editing, methodology. **Lucie Barateau:** writing – review and editing. **Anna Laura Rassu:** writing – review and editing. **Sofiene Chenini:** writing – review and editing. **Isabelle Jaussent:** writing – review and editing, formal analysis, validation, methodology. **Yves Dauvilliers:** supervision, resources, writing – original draft, writing – review and editing, validation, methodology, conceptualization, investigation, funding acquisition.

## Funding

The authors have nothing to report.

## Disclosure

The authors have nothing to report.

## Conflicts of Interest

The authors declare no conflicts of interest.

## Supporting information


**Figure S1:** ROC curves and AUC values for 7am‐Lapses and Delta 7am‐7pm‐Lapses.

**Table S1:** Performance of PVT lapses cut‐offs to identify severe sleep inertia by comparing IH patients with self‐reported severe sleep inertia and OSD patients without sleep inertia.
**Table S2:** AUCs of PVT metrics at 07:00 a.m. and ΔAUC relative to number of lapses (RT ≥ 500 msec) for differentiating the 73 IH patients with severe sleep inertia from the 84 OSD patients without sleep inertia.
**Table S3: Δ** (07:00 a.m.—07:00 p.m.) AUCs and ΔAUCs relative to Number of lapses (RT ≥ 500 msec) for differentiating the 73 IH patients with severe sleep inertia from the 84 patients with other sleep disorders without sleep inertia.
**Table S4:** Clinical and polysomnographic characteristics associated with a reduced mean 1/RT at PVT upon awakening in the whole population*.
**Table S5:** Clinical and polysomnographic characteristics associated with a reduced mean 1/RT at PVT upon awakening in IH patients*.
**Table S6:** Clinical and polysomnographic characteristics associated with a reduced mean 1/RT at PVT upon awakening in OSD patients*.

## Data Availability

The data that support the findings of this study are available on request from the corresponding author. The data are not publicly available due to privacy or ethical restrictions.
