# Supplementary material for: Diagnostic Value of Psychomotor Vigilance Task for Severe Sleep Inertia in Idiopathic Hypersomnia Versus Other Sleep Disorders Without Sleep Inertia
Source: J Sleep Res. 2026 May 31;35(5):e70377. doi: 10.1111/jsr.70377 (PMC13619086; doi:10.1111/jsr.70377)
Supplement: Supplementary file 1 [file JSR-35-e70377-s001.docx]

**Supplementary Table 1:** Performance of PVT lapses cut-offs to identify severe sleep inertia by comparing IH patients with self-reported severe sleep inertia and OSD patients without sleep inertia.

| *Cut Offs* | *AUC_app_ [95%CI] ^(1)^* | *Sensitivity % [95% CI]* | *Specificity % [95% CI]* | *Number of correctly  classified* | *LR+* | *LR-* | *AUC_boot_ [95%CI] ^(2)^* |
| --- | --- | --- | --- | --- | --- | --- | --- |
| 7am-Lapses | *0.777 [0.703-0.851]* |  |  |  |  |  | *0.774 [0.680-0.867]* |
| <1 |  | 95.89 [88.46;99.14] | 25 [16.19;35.64] | 91 | 1.28 | 0.16 |  |
| <4^3^ |  | 72.6 [60.91;82.39] | 69.05 [58.02;78.69] | 111 | 2.35 | 0.4 |  |
| <5 |  | 63.01 [50.91;74.03] | 73.81 [63.07;82.8] | 108 | 2.41 | 0.5 |  |
| <7 |  | 57.53 [45.41;69.03] | 82.14 [72.26;89.65] | 111 | 3.22 | 0.52 |  |
| <10 |  | 50.68 [38.72;62.6] | 90.48 [82.09;95.8] | 113 | 5.32 | 0.55 |  |
| Delta 7am-7pm | *0.737 [0.658-0.816]* |  |  |  |  |  | *0.738 [0.635-0.843]* |
| <1 |  | 80.82 [69.92;89.1] | 57.14 [45.88;67.89] | 107 | 1.89 | 0.34 |  |
| <4^3^ |  | 56.16 [44.05;67.76] | 83.33 [73.62;90.58] | 111 | 3.37 | 0.53 |  |
| <5 |  | 47.95 [36.1;59.96] | 86.9 [77.78;93.28] | 108 | 3.66 | 0.6 |  |
| <7 |  | 39.73 [28.45;51.86] | 92.86 [85.1;97.33] | 107 | 5.56 | 0.65 |  |
| <10 |  | 31.51 [21.13;43.44] | 95.24 [88.25;98.69] | 103 | 6.62 | 0.72 |  |

^1^AUC_app_: apparent area under the curve; ^2^AUC_boot_: bootstrap estimate based on 200 resamples; ^3^Index de Youden

**Supplementary Table 2.** AUCs of PVT metrics at 07:00 am and ΔAUC relative to number of lapses (RT ≥500 msec) for differentiating the 73 IH patients with severe sleep inertia from the 84 OSD patients without sleep inertia

| **Metric** | **AUC  [95%CI]** | **ΔAUC=AUC (metric) – AUC (Number of lapses)  [95%CI]** | **p** |
| --- | --- | --- | --- |
| 07:00 am - Number of lapses | 0.78 [0.70; 0.85] | - |  |
| 07:00 am - Number of lapses + false start | 0.77 [0.70;0.84] | -0.00546 [-0.0111;0.000232] | 0.06 |
| 07:00 am - Mean RT | 0.76 [0.68 ;0.84] | -0.0266 [-0.0552;0.00197] | 0.07 |
| 07:00 am - Median RT | 0.78 [0.70 ;0.85] | -0.00761 [-0.0435;0.0283] | 0.68 |
| 07:00 am - Mean 1/RT | 0.78 [0.71 ;0.86] | -0.00265 [-0.0356;0.0303] | 0.88 |
| 07:00 am - Slowest 10% 1/RT | 0.74 [0.66 ;0.81] | -0.0499 [-0.0803;-0.0196] | 0.001 |
| 07:00 am - Fastest 10% RT | 0.77 [0.70 ;0.85] | -0.0114 [-0.0638;0.0410] | 0.67 |

ΔAUC values represent the difference between each metric’s AUC and the AUC of Number of Lapses (RT ≥500 msec) at 07:00 am

**Supplementary Table 3. Δ** (07:00 am – 07:00 pm) AUCs and ΔAUCs relative to Number of lapses (RT ≥500 msec) for differentiating the 73 IH patients with severe sleep inertia from the 84 patients with other sleep disorders without sleep inertia

| **Metric** | **AUC  [95%CI]** | **ΔAUC = AUC (metric) – AUC (Δ Number of lapses)  [95% CI]** | **p** |
| --- | --- | --- | --- |
| Δ Number of lapses | 0.74 [0.66;0.82] | **-** |  |
| Δ Number of lapses + false start | 0.73 [0.65;0.81] | -0.00711 [-0.0148 ;0.000543] | 0.07 |
| Δ Mean RT | 0.77 [0.69;0.85] | 0.0183 [-0.0482 ; 0.0849] | 0.59 |
| Δ Median RT | 0.80 [0.72;0.87] | 0.0446 [-0.0293 ; 0.1185] | 0.24 |
| Δ Mean 1/RT | 0.76 [0.68;0.84] | 0.00636 [-0.0631 ; 0.0759] | 0.86 |
| Δ Slowest 10% 1/RT | 0.70 [0.61;0.78] | -0.0540 [-0.1213 ; 0.0132] | 0.11 |
| Δ Fastest 10% RT | 0.78 [0.70;0.85] | 0.0233 [-0.0578 ; 0.1043] | 0.57 |

ΔAUC values represent the difference between each Δ metric’s AUC and the AUC of Δ Number of Lapses (RT ≥500 msec).

**Supplementary Table 4.** Clinical and polysomnographic characteristics associated with a reduced mean 1/RT at PVT upon awakening in the whole population*

|  | **7am-mean 1/RT ≥2.60**  N=183 | **7am-mean 1/RT <2.60**  N=166 | *p^1^* | *p^2^* | **Delta 7am-7pm-mean 1/RT**  **≥**-**0.26**  N=165 | **Delta 7am-7pm-mean 1/RT**  <-**0.26**  N=182 | *p^1^* | *p^2^* | *p^3^* |  |
| --- | --- | --- | --- | --- | --- | --- | --- | --- | --- | --- |
| **Demographic and clinical variables** | | | | | | | | | | |
| Age (years) ^a^ | 30.94 (± 11.06) | 30.63 (± 10.88) | 0.79 | 0.56 | 32.39 (± 11.58) | 29.41 (± 10.23) | 0.01 | - | - |  |
| Sex, female ^b^ | 103 (56.28) | 123 (74.10) | 0.0006 | - | 97 (58.79) | 127 (69.78) | 0.03 | - | - |  |
| BMI (kg/m^2^) ^a^ | 24.38 (± 5.02) | 23.54 (± 4.49) | 0.10 | 0.30 | 24.36 (± 5.10) | 23.64 (± 4.48) | 0.17 | 0.64 | 0.69 |  |
| Diagnosis ^b^ |  |  |  |  |  |  |  |  |  |  |
| IH | 55 (30.05) | 75 (45.18) | 0.004 | - | 49 (29.70) | 81 (44.51) | 0.005 | - | - |  |
| Other sleep disorders | 128 (69.95) | 91 (54.82) |  |  | 116 (70.30) | 101 (55.49) |  |  |  |  |
| ESS score ^a^ | 13.71 (± 5.34) | 15.30 (± 4.69) | 0.004 | 0.02 | 14.43 (± 5.39) | 14.46 (± 4.84) | 0.95 | 0.80 | 0.92 |  |
| ESS score, >10 ^b^ | 129 (71.67) | 142 (86.06) | 0.001 | 0.009 | 127 (77.91) | 142 (78.89) | 0.83 | 0.70 | 0.97 |  |
| Total IHSS score ^a^ | 27.36 (± 9.15) | 32.88 (± 7.11) | <0.0001 | <0.0001 | 28.45 (± 9.24) | 31.43 (± 7.87) | 0.003 | 0.04 | 0.008 |  |
| Total IHSS score, ≥ 26 ^b^ | 101 (62.35) | 138 (86.79) | <0.0001 | <0.0001 | 99 (66.89) | 138 (80.70) | 0.005 | 0.04 | 0.01 |  |
| Partial IHSS score ^a^ | 23.72 (± 7.58) | 27.60 (± 5.94) | <0.0001 | <0.0001 | 24.79 (± 7.58) | 26.27 (± 6.54) | 0.06 | 0.35 | 0.17 |  |
| Self-reported sleep inertia ^b^ |  |  |  |  |  |  |  |  |  |  |
| Absent | 78 (42.62) | 26 (15.66) | <0.0001 | 0.0001 | 75 (45.45) | 28 (15.38) | <0.0001 | <0.0001 | <0.0001 |  |
| Mild | 43 (23.50) | 40 (24.10) |  |  | 36 (21.82) | 46 (25.27) |  |  |  |  |
| Severe without drunkenness | 42 (22.95) | 57 (34.34) |  |  | 36 (21.82) | 63 (34.62) |  |  |  |  |
| Severe with drunkenness | 20 (10.93) | 43 (25.90) |  |  | 18 (10.91) | 45 (24.73) |  |  |  |  |
| Driving impairment, Moderate to very significant ^b^ | 68 (37.16) | 79 (47.59) | 0.05 | 0.11 | 66 (40.00) | 80 (43.96) | 0.46 | 0.63 | 0.40 |  |
| Night-time sleep needs, ≥ 9 hours ^b^ | 92 (57.14) | 116 (74.84) | 0.001 | 0.02 | 83 (56.46) | 123 (73.65) | 0.002 | 0.08 | 0.05 |  |
| Habitual bedtime (hh:mm) ^a^ | 23:10 (±0:58) | 22:49 (±1:06) | 0.007 | 0.01 | 22:59 (±1:04) | 22:59 (±1:02) | 0.95 | 0.79 | 0.55 |  |
| Habitual wake up time (hh:mm) ^a^ | 7:55 (±1:49) | 8:12 (±1:52) | 0.21 | 0.24 | 7:54 (±1:41) | 8:10 (±1:55) | 0.25 | 0.64 | 0.52 |  |
| BDI-II score ^a^ | 12.88 (± 8.97) | 15.06 (± 10.13) | 0.05 | 0.08 | 13.93 (± 9.08) | 13.99 (± 10.15) | 0.96 | 0.93 | 0.78 |  |
| BDI-II score ^b^ |  |  |  |  |  |  |  |  |  |  |
| <14 | 84 (57.93) | 73 (48.99) | 0.12 | 0.11 | 72 (52.55) | 85 (54.84) | 0.93 | 0.92 | 0.97 |  |
| 14-20 | 21 (14.48) | 35 (23.49) |  |  | 26 (18.98) | 28 (18.06) |  |  |  |  |
| ≥ 20 | 40 (27.59) | 41 (27.52) |  |  | 39 (28.47) | 42 (27.10) |  |  |  |  |
| 7:00-KSS score ^a^ | 5.73 (± 1.99) | 6.91 (± 1.94) | <0.0001 | <0.0001 | 5.67 (± 1.96) | 6.88 (± 1.95) | <0.0001 | <0.0001 | <0.0001 |  |
| **Polysomnography** | | | | | | | | | | |
| Sleep duration (min) ^a^ | 424.59 (± 55.38) | 425.97 (± 55.69) | 0.82 | 0.36 | 422.52 (± 59.05) | 428.41 (± 51.65) | 0.32 | 0.70 | 0.70 |  |
| Sleep efficiency (%) ^a^ | 84.39 (± 10.45) | 84.63 (± 9.73) | 0.83 | 0.31 | 83.17 (± 11.19) | 85.86 (± 8.76) | 0.01 | 0.27 | 0.30 |  |
| Sleep latency (min) ^a^ | 17.81 (± 26.36) | 21.58 (± 61.26) | 0.47 | 0.51 | 17.24 (± 27.29) | 21.92 (± 58.64) | 0.39 | 0.37 | 0.39 |  |
| REM sleep latency (min) ^a^ | 88.39 (± 59.43) | 88.57 (± 56.48) | 0.98 | 0.78 | 84.69 (± 61.39) | 92.51 (± 54.50) | 0.21 | 0.24 | 0.26 |  |
| WASO (min) ^a^ | 54.80 (± 42.09) | 55.57 (± 46.25) | 0.87 | 0.16 | 59.66 (± 48.09) | 50.20 (± 38.79) | 0.05 | 0.56 | 0.57 |  |
| N1 (%) ^a^ | 5.42 (± 4.08) | 5.76 (± 5.50) | 0.51 | 0.08 | 6.12 (± 5.17) | 5.02 (± 4.31) | 0.04 | 0.30 | 0.33 |  |
| N2 (%) ^a^ | 53.85 (± 8.39) | 53.59 (± 8.26) | 0.78 | 0.71 | 52.82 (± 8.76) | 54.53 (± 7.85) | 0.06 | 0.07 | 0.08 |  |
| N3 (%) ^a^ | 19.29 (± 5.93) | 19.15 (± 6.75) | 0.84 | 0.40 | 19.14 (± 6.28) | 19.32 (± 6.38) | 0.79 | 0.69 | 0.63 |  |
| REM (%) ^a^ | 21.39 (± 6.36) | 21.44 (± 5.44) | 0.93 | 0.97 | 21.88 (± 6.13) | 21.06 (± 5.68) | 0.20 | 0.16 | 0.21 |  |
| PLMS index (/hour) ^a^ | 7.72 (± 14.37) | 7.75 (± 17.10) | 0.99 | 0.51 | 9.26 (± 17.18) | 6.28 (± 14.18) | 0.08 | 0.38 | 0.38 |  |
| AHI (/hour) ^a^ | 6.03 (± 10.68) | 3.72 (± 7.10) | 0.03 | 0.34 | 5.97 (± 11.13) | 3.89 (± 6.82) | 0.05 | 0.53 | 0.44 |  |
| Micro-arousal index (/hour) ^a^ | 13.73 (± 9.74) | 12.51 (± 7.76) | 0.20 | 0.74 | 14.47 (± 9.93) | 11.83 (± 7.40) | 0.007 | 0.13 | 0.15 |  |
| Last hour N1 (%) ^a^ | 6.77 (± 6.94) | 5.91 (± 5.70) | 0.44 | 0.83 | 7.75 (± 8.05) | 5.37 (± 4.85) | 0.05 | 0.31 | 0.31 |  |
| Last hour N2 (%) ^a^ | 43.44 (± 24.04) | 43.64 (± 26.36) | 0.96 | 0.88 | 40.80 (± 27.20) | 45.65 (± 23.53) | 0.28 | 0.50 | 0.48 |  |
| Last hour N3 (%) ^a^ | 6.28 (± 12.49) | 3.26 (± 9.21) | 0.13 | 0.13 | 4.25 (± 8.98) | 5.16 (± 12.25) | 0.65 | 0.59 | 0.61 |  |
| Last hour REM (%) ^a^ | 29.32 (± 22.51) | 28.88 (± 22.62) | 0.91 | 0.70 | 28.27 (± 22.67) | 30.36 (± 22.26) | 0.60 | 0.97 | 0.98 |  |
| Last hour WAKE (%) ^a^ | 14.45 (± 19.60) | 18.48 (± 25.63) | 0.32 | 0.19 | 19.17 (± 24.37) | 13.66 (± 20.20) | 0.17 | 0.46 | 0.45 |  |
| **MSLT** | | | | | | | | | | |
| MSL on MSLT (min) ^a^ | 9.53 (± 4.76) | 10.61 (± 5.00) | 0.06 | 0.01 | 9.44 (± 4.77) | 10.70 (± 4.94) | 0.03 | 0.001 | 0.0007 |  |
| Total SOREMPS on MSLT/PSG ^a^ | 1.04 (± 1.92) | 0.66 (± 1.59) | 0.06 | 0.27 | 1.18 (± 2.00) | 0.54 (± 1.47) | 0.002 | 0.009 | 0.01 |  |

* One patient was excluded from the 7am-mean 1/RT and three from the Delta 7am-7pm-mean 1/RT analyses due to RTs ≥500 msec.

^a^ Quantitative variables are expressed as means (± standard deviation). ^b^ Categorical variables are shown as n (%).

^1^ Crude association; ^2^ Adjustment for sex, age and diagnosis; ^3^ Adjustment for sex, age, diagnosis and the number of PVT lapses at 7:00 pm.

AHI = apnea-hypopnea index, BDI-II = Beck Depression Inventory II, BMI = body mass index, ESS = Epworth Sleepiness Scale, IHSS = Idiopathic Hypersomnia Severity Scale, KSS = Karolinska Sleepiness Scale, MSL = mean sleep latency, MSLT = multiple sleep latency test, N1 = sleep stage N1, N2 = sleep stage N2, N3 = sleep stage N3, PLMS = periodic limb movements during sleep, REM = rapid eye movement, WASO = wake after sleep onset.

**Supplementary Table 5.** Clinical and polysomnographic characteristics associated with a reduced mean 1/RT at PVT upon awakening in IH patients*

|  | **7am-mean 1/RT ≥2.60**  N=55 | **7am-mean 1/RT <2.60**  N=75 | *p^1^* | *p^2^* | **Delta 7am-7pm-mean 1/RT**  **≥**-**0.26**  N=49 | **Delta 7am-7pm-mean 1/RT**  <-**0.26**  N=81 | *p^1^* | *p^3^* |
| --- | --- | --- | --- | --- | --- | --- | --- | --- |
| **Demographic and clinical variables** | | | | | | | | |
| Age (years) ^a^ | 26.15 (± 6.62) | 29.08 (± 10.24) | 0.07 | - | 30.41 (± 9.98) | 26.29 (± 7.98) | 0.01 | - |
| Sex, female ^b^ | 42 (76.36) | 65 (86.67) | 0.13 | 0.12 | 42 (85.71) | 65 (80.25) | 0.43 | 0.42 |
| BMI (kg/m^2) a^ | 22.61 (± 3.78) | 22.92 (± 4.51) | 0.68 | 0.80 | 22.77 (± 4.58) | 22.81 (± 3.99) | 0.96 | 0.77 |
| ESS score ^a^ | 14.59 (± 4.19) | 15.99 (± 4.10) | 0.06 | 0.14 | 16.02 (± 4.00) | 15.03 (± 4.27) | 0.19 | 0.48 |
| ESS score, >10 ^b^ | 46 (85.19) | 68 (90.67) | 0.34 | 0.42 | 46 (93.88) | 68 (85.00) | 0.14 | 0.19 |
| Total IHSS score ^a^ | 30.74 (± 6.72) | 34.69 (± 7.42) | 0.005 | 0.003 | 31.52 (± 7.83) | 33.95 (± 7.00) | 0.08 | 0.08 |
| Total IHSS score, ≥ 26 ^b^ | 39 (78.00) | 66 (91.67) | 0.04 | 0.02 | 35 (79.55) | 70 (89.74) | 0.12 | 0.18 |
| Partial IHSS score ^a^ | 26.24 (± 5.27) | 29.11 (± 5.82) | 0.008 | 0.007 | 27.30 (± 5.96) | 28.29 (± 5.64) | 0.36 | 0.31 |
| Self-reported sleep inertia ^b^ |  |  |  |  |  |  |  |  |
| Absent | 11 (20.00) | 9 (12.00) | 0.05 | 0.03 | 15 (30.61) | 5 (6.17) | 0.006 | 0.01 |
| Mild | 20 (36.36) | 18 (24.00) |  |  | 14 (28.57) | 24 (29.63) |  |  |
| Severe without drunkenness | 16 (29.09) | 21 (28.00) |  |  | 10 (20.41) | 27 (33.33) |  |  |
| Severe with drunkenness | 8 (14.55) | 27 (36.00) |  |  | 10 (20.41) | 25 (30.86) |  |  |
| Driving impairment, Moderate to very significant ^b^ | 28 (50.91) | 41 (54.67) | 0.67 | 0.77 | 28 (57.14) | 41 (50.62) | 0.47 | 0.59 |
| Night-time sleep needs, ≥ 9 hours ^b^ | 38 (76.00) | 61 (85.92) | 0.17 | 0.06 | 33 (75.00) | 66 (85.71) | 0.15 | 0.35 |
| Habitual bedtime (hh:mm) ^a^ | 23:14 (±0:59) | 22:54 (±1:01) | 0.07 | 0.17 | 22:58 (±1:04) | 23:05 (±0:59) | 0.52 | 0.96 |
| Habitual wake up time (hh:mm) ^a^ | 8:11 (±1:53) | 8:16 (±1:56) | 0.81 | 0.35 | 8:00 (±1:46) | 8:22 (±1:59) | 0.29 | 0.78 |
| BDI-II score ^a^ | 11.85 (± 8.62) | 15.49 (± 11.07) | 0.05 | 0.07 | 12.55 (± 9.63) | 14.86 (± 10.57) | 0.22 | 0.15 |
| BDI-II score ^b^ |  |  |  |  |  |  |  |  |
| <14 | 32 (60.38) | 38 (51.35) | 0.50 | 0.54 | 30 (61.22) | 40 (51.28) | 0.51 | 0.46 |
| 14-20 | 7 (13.21) | 15 (20.27) |  |  | 8 (16.33) | 14 (17.95) |  |  |
| ≥ 20 | 14 (26.42) | 21 (28.38) |  |  | 11 (22.45) | 24 (30.77) |  |  |
| 7:00-KSS score ^a^ | 6.33 (± 1.95) | 7.29 (± 1.90) | 0.009 | 0.009 | 6.21 (± 2.11) | 7.32 (± 1.77) | 0.003 | 0.002 |
| **Polysomnography** | | | | | | | | |
| Sleep duration (min) ^a^ | 447.89 (± 39.16) | 446.57 (± 38.39) | 0.85 | 0.86 | 444.93 (± 41.66) | 448.49 (± 36.72) | 0.61 | 0.95 |
| Sleep efficiency (%) ^a^ | 89.36 (± 5.53) | 88.71 (± 5.98) | 0.53 | 0.86 | 88.69 (± 5.78) | 89.17 (± 5.81) | 0.65 | 0.87 |
| Sleep latency (min) ^a^ | 15.19 (± 13.99) | 23.17 (± 88.54) | 0.55 | 0.51 | 13.82 (± 13.26) | 23.40 (± 85.25) | 0.56 | 0.60 |
| REM sleep latency (min) ^a^ | 100.88 (± 53.11) | 93.64 (± 47.97) | 0.42 | 0.37 | 89.99 (± 45.59) | 100.76 (± 52.55) | 0.24 | 0.18 |
| WASO (min) ^a^ | 33.92 (± 24.30) | 39.65 (± 29.65) | 0.24 | 0.49 | 35.96 (± 26.41) | 37.99 (± 28.37) | 0.68 | 0.22 |
| N1 (%) ^a^ | 3.90 (± 2.44) | 4.20 (± 3.10) | 0.54 | 0.81 | 4.19 (± 2.89) | 4.00 (± 2.82) | 0.72 | 0.87 |
| N2 (%) ^a^ | 55.39 (± 6.16) | 53.78 (± 7.64) | 0.20 | 0.17 | 54.25 (± 6.49) | 54.59 (± 7.44) | 0.79 | 0.74 |
| N3 (%) ^a^ | 19.57 (± 4.64) | 19.76 (± 6.93) | 0.85 | 0.64 | 19.80 (± 5.33) | 19.61 (± 6.48) | 0.86 | 0.59 |
| REM (%) ^a^ | 21.09 (± 4.49) | 22.21 (± 5.13) | 0.20 | 0.20 | 21.73 (± 4.08) | 21.74 (± 5.34) | 0.99 | 0.95 |
| PLMS index (/hour) ^a^ | 4.67 (± 8.19) | 5.10 (± 9.15) | 0.78 | 0.97 | 6.32 (± 9.17) | 4.06 (± 8.39) | 0.16 | 0.26 |
| AHI (/hour) ^a^ | 2.77 (± 3.43) | 2.09 (± 2.66) | 0.22 | 0.10 | 2.88 (± 3.59) | 2.07 (± 2.58) | 0.14 | 0.28 |
| Micro-arousal index (/hour) ^a^ | 10.79 (± 6.52) | 9.93 (± 5.41) | 0.41 | 0.28 | 11.87 (± 7.22) | 9.34 (± 4.73) | 0.02 | 0.05 |
| Last hour N1 (%) ^a^ | 4.23 (± 3.99) | 4.28 (± 4.72) | 0.96 | 0.72 | 4.17 (± 4.76) | 4.30 (± 4.27) | 0.91 | 0.46 |
| Last hour N2 (%) ^a^ | 44.05 (± 21.10) | 48.24 (± 23.87) | 0.46 | 0.43 | 41.21 (± 23.43) | 48.76 (± 22.15) | 0.22 | 0.22 |
| Last hour N3 (%) ^a^ | 4.70 (± 11.01) | 3.11 (± 10.14) | 0.55 | 0.65 | 4.54 (± 10.81) | 3.46 (± 10.42) | 0.70 | 0.51 |
| Last hour REM (%) ^a^ | 34.49 (± 21.62) | 32.23 (± 22.26) | 0.68 | 0.92 | 33.58 (± 23.09) | 33.04 (± 21.54) | 0.93 | 0.46 |
| Last hour WAKE (%) ^a^ | 12.92 (± 18.99) | 12.23 (± 22.07) | 0.89 | 0.58 | 16.83 (± 27.94) | 10.61 (± 16.45) | 0.27 | 0.66 |
| **MSLT (N=130)** | | | | | | | | |
| MSL on MSLT (min) ^a^ | 7.73 (± 3.18) | 9.00 (± 4.25) | 0.07 | 0.05 | 7.85 (± 3.48) | 8.84 (± 4.06) | 0.16 | 0.15 |
| MSL on MSLT, ≤8 min ^b^ | 31 (56.36) | 35 (46.67) | 0.28 | 0.21 | 25 (51.02) | 41 (50.62) | 0.96 | 0.89 |
| Total SOREMPS on MSLT/PSG ^a^ | 0.11 (± 0.37) | 0.15 (± 0.56) | 0.67 | 0.59 | 0.10 (± 0.35) | 0.16 (± 0.61) | 0.61 | 0.73 |
| **Bed rest (N=120)** | | | | | | | | |
| 32-h bed rest TST (hour) ^a^ | 21.03 (± 2.94) | 21.16 (± 2.62) | 0.81 | 0.46 | 20.74 (± 3.02) | 21.34 (± 2.55) | 0.25 | 0.67 |
| 32-h bed rest TST, ≥19 hours ^b^ | 42 (84.00) | 63 (90.00) | 0.33 | 0.15 | 36 (76.60) | 69 (94.52) | 0.007 | 0.03 |

* One patient was excluded from the 7am-mean 1/RT and one from Delta 7am-7pm-mean 1/RT analyses due to RTs ≥500 msec.

^a^ Quantitative variables are expressed as means (± standard deviation). ^b^ Categorical variables are shown as n (%).

^1^ Crude association; ^2^ Adjustment for the number of PVT lapses at 7:00 pm.

^1^ Crude association; ^2^ Adjustment for age; ^3^ Adjustment for age and the number of PVT lapses at 7:00 pm.

AHI = apnea-hypopnea index, BDI-II = Beck Depression Inventory II, BMI = body mass index, ESS = Epworth Sleepiness Scale, IHSS = Idiopathic Hypersomnia Severity Scale, KSS = Karolinska Sleepiness Scale, MSL = mean sleep latency, MSLT = multiple sleep latency test, N1 = sleep stage N1, N2 = sleep stage N2, N3 = sleep stage N3, PLMS = periodic limb movements during sleep, REM = rapid eye movement, TST = total sleep time, WASO = wake after sleep onset.

**Supplementary Table 6.** Clinical and polysomnographic characteristics associated with a reduced mean 1/RT at PVT upon awakening in OSD patients*

|  | **7:00 am-mean 1/RT ≥2.60**  N=128 | **7:00 am-mean 1/RT <2.60**  N=91 | ***p^1^*** | ***p^(2^*** | **Delta 7am-7pm-mean 1/RT**  **≥**-**0.26**  N=116 | **Delta 7am-7pm-mean 1/RT**  <-**0.26**  N=101 | ***p^1^*** | ***p^2^*** | ***p^3^*** |  |
| --- | --- | --- | --- | --- | --- | --- | --- | --- | --- | --- |
| **Demographic and clinical variables** | | | | | | | | | | |
| Age (years) ^a^ | 33.00 (± 11.94) | 31.91 (± 11.27) | 0.50 | 0.87 | 33.23 (± 12.14) | 31.92 (± 11.14) | 0.41 | 0.27 | 0.48 |  |
| Sex, female ^b^ | 61 (47.66) | 58 (63.74) | 0.02 | - | 55 (47.41) | 62 (61.39) | 0.04 | - | - |  |
| BMI (kg/m^2^) ^a^ | 25.14 (± 5.30) | 24.05 (± 4.43) | 0.11 | 0.36 | 25.03 (± 5.18) | 24.31 (± 4.74) | 0.29 | 0.65 | 0.82 |  |
| Treatment status, Naïve ^b^ | 103 (80.47) | 66 (72.53) | 0.17 | 0.08 | 89 (76.72) | 79 (78.22) | 0.79 | 0.82 | 0.68 |  |
| Diagnosis ^b^ |  |  | 0.04 | - |  |  | 0.03 | - | - |  |
| NSH | 34 (26.56) | 41 (45.05) |  |  | 34 (29.31) | 41 (40.59) |  |  |  |  |
| NT1 | 37 (28.91) | 17 (18.68) |  |  | 37 (31.90) | 15 (14.85) |  |  |  |  |
| OSAS | 32 (25.00) | 18 (19.78) |  |  | 25 (21.55) | 25 (24.75) |  |  |  |  |
| Other | 25 (19.53) | 15 (16.48) |  |  | 20 (17.24) | 20 (19.80) |  |  |  |  |
| ESS score ^a^ | 13.33 (± 5.73) | 14.72 (± 5.08) | 0.07 | 0.03 | 13.75 (± 5.77) | 14.01 (± 5.23) | 0.73 | 0.13 | 0.05 |  |
| ESS score, >10 ^b^ | 83 (65.87) | 74 (82.22) | 0.009 | 0.005 | 81 (71.05) | 74 (74.00) | 0.63 | 0.18 | 0.07 |  |
| Total IHSS score ^a^ | 25.86 (± 9.70) | 31.38 (± 6.51) | <0.0001 | 0.0003 | 27.15 (± 9.51) | 29.31 (± 7.97) | 0.09 | 0.25 | 0.09 |  |
| Total IHSS score, ≥ 26 ^b^ | 62 (55.36) | 72 (82.76) | <0.0001 | 0.0006 | 64 (61.54) | 68 (73.12) | 0.09 | 0.17 | 0.06 |  |
| Partial IHSS score ^a^ | 22.59 (± 8.17) | 26.34 (± 5.77) | 0.0007 | 0.005 | 23.73 (± 7.96) | 24.55 (± 6.78) | 0.44 | 0.77 | 0.47 |  |
| Self-reported sleep inertia ^b^ |  |  | <0.0001 | 0.001 |  |  | 0.001 | 0.0008 | 0.0001 |  |
| Absent | 67 (52.34) | 17 (18.68) |  |  | 60 (51.72) | 23 (22.77) |  |  |  |  |
| Mild | 23 (17.97) | 22 (24.18) |  |  | 22 (18.97) | 22 (21.78) |  |  |  |  |
| Severe without drunkenness | 26 (20.31) | 36 (39.56) |  |  | 26 (22.41) | 36 (35.64) |  |  |  |  |
| Severe with drunkenness | 12 (9.38) | 16 (17.58) |  |  | 8 (6.90) | 20 (19.80) |  |  |  |  |
| Driving impairment, Moderate to very significant ^b^ | 40 (31.25) | 38 (41.76) | 0.11 | 0.07 | 38 (32.76) | 39 (38.61) | 0.37 | 0.13 | 0.05 |  |
| Night-time sleep needs, ≥ 9 hours ^b^ | 54 (48.65) | 55 (65.48) | 0.02 | 0.12 | 50 (48.54) | 57 (63.33) | 0.04 | 0.19 | 0.19 |  |
| Habitual bedtime (hh:mm) ^a^ | 23:08 (±0:58) | 22:43 (±1:12) | 0.14 | 0.07 | 23:01 (±1:05) | 22:53 (± 8.72) | 0.39 | 0.14 | 0.06 |  |
| Habitual wake up time (hh:mm) ^a^ | 7:43 (±1:44) | 8:07 (±1:49) | 0.19 | 0.32 | 7:51 (±1:38) | 7:55 (±1:49) | 0.81 | 0.63 | 0.68 |  |
| BDI-II score ^a^ | 13.47 (± 9.16) | 14.64 (± 9.15) | 0.41 | 0.65 | 14.70 (± 8.72) | 13.12 (± 9.69) | 0.27 | 0.22 | 0.34 |  |
| BDI-II score ^b^ |  |  | 0.18 | 0.10 |  |  | 0.36 | 0.29 | 0.33 |  |
| <14 | 52 (56.52) | 35 (46.67) |  |  | 42 (47.73) | 45 (58.44) |  |  |  |  |
| 14-20 | 14 (15.22) | 20 (26.67) |  |  | 18 (20.45) | 14 (18.18) |  |  |  |  |
| ≥ 20 | 26 (28.26) | 20 (26.67) |  |  | 28 (31.82) | 18 (23.38) |  |  |  |  |
| 7:00-KSS score ^a^ | 5.48 (± 1.96) | 6.60 (± 1.93) | 0.0001 | 0.0009 | 5.44 (± 1.86) | 6.55 (± 2.03) | 0.0001 | 0.002 | 0.0004 |  |
| **Polysomnography** | | | | | | | | | | |
| Sleep duration (min) ^a^ | 414.76 (± 58.34) | 409.04 (± 61.88) | 0.49 | 0.22 | 412.98 (± 62.82) | 412.70 (± 56.18) | 0.97 | 0.79 | 0.95 |  |
| Sleep efficiency (%) ^a^ | 82.26 (± 11.33) | 81.27 (± 10.90) | 0.51 | 0.16 | 80.84 (± 12.08) | 83.21 (± 9.80) | 0.12 | 0.13 | 0.26 |  |
| Sleep latency (min) ^a^ | 18.93 (± 30.14) | 20.27 (± 20.74) | 0.71 | 0.92 | 18.68 (± 31.33) | 20.72 (± 20.14) | 0.58 | 0.84 | 0.90 |  |
| REM sleep latency (min) ^a^ | 82.98 (± 61.38) | 84.39 (± 62.58) | 0.87 | 0.47 | 82.43 (± 67.05) | 85.89 (± 55.39) | 0.68 | 0.23 | 0.20 |  |
| WASO (min) ^a^ | 63.77 (± 44.92) | 68.68 (± 53.04) | 0.46 | 0.09 | 69.67 (± 51.64) | 59.98 (± 43.15) | 0.14 | 0.39 | 0.54 |  |
| N1 (%) ^a^ | 6.08 (± 4.46) | 7.05 (± 6.62) | 0.20 | 0.02 | 6.94 (± 5.68) | 5.84 (± 5.08) | 0.15 | 0.61 | 0.81 |  |
| N2 (%) ^a^ | 53.18 (± 9.13) | 53.44 (± 8.77) | 0.83 | 0.82 | 52.22 (± 9.52) | 54.48 (± 8.20) | 0.07 | 0.35 | 0.43 |  |
| N3 (%) ^a^ | 19.17 (± 6.42) | 18.65 (± 6.59) | 0.56 | 0.14 | 18.86 (± 6.64) | 19.10 (± 6.33) | 0.79 | 0.75 | 0.65 |  |
| REM (%) ^a^ | 21.52 (± 7.02) | 20.81 (± 5.63) | 0.43 | 0.78 | 21.94 (± 6.83) | 20.52 (± 5.92) | 0.11 | 0.58 | 0.66 |  |
| PLMS index (/hour) ^a^ | 9.03 (± 16.18) | 9.93 (± 21.36) | 0.72 | 0.12 | 10.51 (± 19.50) | 8.06 (± 17.34) | 0.34 | 0.71 | 0.60 |  |
| AHI (/hour) ^a^ | 7.40 (± 12.30) | 5.21 (± 9.16) | 0.16 | 0.76 | 7.39 (± 12.87) | 5.33 (± 8.59) | 0.18 | 0.29 | 0.26 |  |
| Micro-arousal index (/hour) ^a^ | 14.99 (± 10.60) | 14.64 (± 8.73) | 0.79 | 0.57 | 15.56 (± 10.72) | 13.83 (± 8.49) | 0.20 | 0.29 | 0.39 |  |
| Last hour N1 (%) ^a^ | 8.75 (± 8.07) | 7.92 (± 6.22) | 0.64 | 0.49 | 10.14 (± 8.94) | 6.79 (± 5.25) | 0.08 | 0.47 | 0.44 |  |
| Last hour N2 (%) ^a^ | 42.96 (± 26.39) | 37.97 (± 28.53) | 0.46 | 0.06 | 40.53 (± 29.84) | 41.54 (± 24.98) | 0.88 | 0.36 | 0.38 |  |
| Last hour N3 (%) ^a^ | 7.50 (± 13.56) | 3.45 (± 8.09) | 0.17 | 0.25 | 4.06 (± 7.72) | 7.40 (± 14.17) | 0.26 | 0.08 | 0.11 |  |
| Last hour REM (%) ^a^ | 25.30 (± 22.65) | 24.75 (± 22.74) | 0.92 | 0.90 | 24.72 (± 22.06) | 26.81 (± 23.02) | 0.71 | 0.76 | 0.82 |  |
| Last hour WAKE (%) ^a^ | 15.65 (± 20.25) | 26.19 (± 27.91) | 0.09 | 0.02 | 20.72 (± 22.05) | 17.70 (± 23.96) | 0.60 | 0.99 | 0.84 |  |
| **MSLT (N=176)** | | | | | | | | | | |
| MSL on MSLT (min) ^a^ | 10.55 (± 5.19) | 12.14 (± 5.20) | 0.005 | 0.70 | 10.29 (± 5.15) | 12.53 (± 5.05) | 0.005 | 0.56 | 0.40 |  |
| Total SOREMPS on MSLT/PSG ^a^ | 1.57 (± 2.22) | 1.14 (± 2.04) | 0.19 | 0.10 | 1.75 (± 2.27) | 0.92 (± 1.92) | 0.01 | 0.59 | 0.53 |  |

* Two patients were excluded from the Delta 7am-7pm-mean 1/RT analysis due to RTs ≥500 msec.

^a^ Quantitative variables are expressed as means (± standard deviation). ^b^ Categorical variables are shown as n (%).

^1^ Crude association; ^2^ Adjustment for sex and diagnosis; ^3^ Adjustment for sex, diagnosis and the number of PVT lapses at 7:00 pm.

AHI = apnea-hypopnea index, BDI-II = Beck Depression Inventory II, BMI = body mass index, ESS = Epworth Sleepiness Scale, IHSS = Idiopathic Hypersomnia Severity Scale, KSS = Karolinska Sleepiness Scale, MSL = mean sleep latency, MSLT = multiple sleep latency test, N1 = sleep stage N1, N2 = sleep stage N2, N3 = sleep stage N3, PLMS = periodic limb movements during sleep, REM = rapid eye movement, WASO = wake after sleep onset.
